# The polychoric dual-component wealth index as an alternative to the DHS index: Addressing the urban bias

**DOI:** 10.7189/jogh.11.04003

**Published:** 2021-01-30

**Authors:** Pierre Martel, Francisco Mbofana, Simon Cousens

**Affiliations:** 1 Multiple Indicator Cluster Survey, Mokhotlong, Lesotho; 2 Ministry of Health, Maputo, Mozambique; 3 London School of Hygiene and Tropical Medicine, London, UK

## Abstract

**Background:**

The DHS wealth index − based on a statistical technique known as principal component analysis − is used extensively in mainstream surveys and epidemiological studies to assign individuals to wealth categories from information collected on common assets and household characteristics. Since its development in the late nineties, the index has established itself as a standard and, due to its ease of use, has led to a large and welcome increase in the analysis of inequalities. The index is, however, known to present some serious limitations, one being a bias towards patterns of urban wealth: the so-called “urban bias”.

**Methods:**

We use 10 data sets − 5 MICS (Multiple Indicator Cluster Survey), 4 DHS (Demographic and Health Survey) and one HBS (Household Budget Survey) − to demonstrate that urban bias continues to be a prominent and worrying feature of the wealth index, even after several methodological changes implemented in recent years to try to reduce it. We then propose and investigate an approach to improve the performance of the index and reduce the urban bias. This approach involves the use of ordinal rather than dummy variables, of a polychoric instead of a product-moment correlation matrix, and the use of two principal components rather than one. These approaches are used jointly to produce the polychoric dual-component wealth index (P2C).

**Results:**

The P2C index enables a larger proportion of the variance of the asset variables to be accounted for, results in all assets contributing positively to the wealth score, exploits added analytical power from ordinal variables, and incorporates the extra dimension of wealth expressed by the second principal component. It results in a better representation of typically rural characteristics of wealth and leads to the identification of more plausible distributions of both the urban and rural populations across wealth quintiles, which are closer to expenditure quintiles than the standard DHS index.

**Conclusions:**

The P2C wealth index can be easily applied to mainstream surveys, such as the MICS and DHS, and to epidemiological studies; it yields more credible distributions of rural and urban subpopulations across wealth quintiles. It is proposed as an alternative to the DHS wealth index.

## Equity

Since the dawn of the 21^st^ century, equity has received ever increasing attention from public institutions, and the programmes of most development agencies are now characterized by a strong equity focus. For example, in 2010 UNICEF embraced a renewed focus on issues of equity and adjusted its organizational efforts to address the poorest and most underserved children and communities, considering this strategically right both in principle and in practice [1]; its current slogan “for every child” testifies that such is still its position today. The World Bank holds that no society can achieve its potential or meet the vast challenges of the 21st century without the full and equal participation of all its people [2], and focuses explicitly on the condition of the “bottom 40%” of the populations. The *Countdown to 2015* motivated intense scrutiny on equity in health [3,4] and in other sectors. Above all, perhaps, the sustainable development goals (SDGs) on which all countries of the world are currently focused amply demonstrate the central position that the international community currently gives to equity in development [5].

## DHS index

While the multifaceted nature of poverty cannot be denied, the analysis of service utilization and development outcomes by categories of material wealth in diverse populations and groups is an important step in assessing equity. This obviously requires a valid measure of socioeconomic status (SES). Collecting reliable data on household income or expenditure, as a measure of SES, is time consuming and can be difficult in some settings [6-9]. Further, such burdensome methods do not lend themselves to easy inclusion in most surveys. In 1998, Filmer and Pritchett developed an approach to calculating a wealth index [10,11] based on a list of assets and household characteristics. The index is widely known as the “DHS wealth index” and will be referred to as such in this article. After its conceptualization, the DHS index was rapidly adopted by mainstream surveys such as the Demographic Health Survey (DHS) [12] and the Multiple Indicator Cluster Survey (MICS) [13]. It has also been used in many epidemiological studies, as well as national and sub-national surveys as measure of SES, leading to a large and welcome increase in the analysis of inequalities. While other methodologies have been proposed [9,14,15], some showing performances similar to the DHS index, the latter received such strong institutional support that it has become a standard. Several studies have compared the DHS index to conventional measures of income and consumption expenditure [12,14,16-21]; most show agreement to be moderate at best. A recent review by Poirier et al. [9] concludes that wealth indices based on household assets are measures of household SES that are “valid but distinct” from income and consumption measures. It is further argued that a wealth index based on assets acquired over time rather than on current, sometime highly variable, income/expenditure, may well better relate to behaviour patterns established over years, which is what household surveys often try to measure [9].

## PCA

The construction of the DHS index is based on a statistical technique known as principal component analysis (PCA) [22-24]. PCA is generally regarded as a data reduction technique, permitting the reduction of often large sets of correlated variables into a smaller number of independent variables. This can help to identify patterns of association that may not be easily discernible in the original set of variables. The technique is commonly used in such areas as market research, genetic mapping, face recognition and image compression where the objective is to extract basic patterns from a multiplicity of correlated variables [22,23]. The first principal component typically captures a relatively small proportion of the total variance of the variables being analysed and the goal is generally to find the smallest possible set of principal components that captures an adequate proportion of total variation. Thus, PCA is most useful when variables being analysed are strongly correlated with each other [8,21,22], and when the first few principal components capture a sizeable proportion of the total variance.

## Is the first principal component of the PCA sufficient?

The DHS index relies, however, exclusively on the first principal component of the PCA based on which each household is allocated a score; household members are then assigned to wealth categories, usually quintiles, as per the score of the household in which they live. Using an approach such as PCA to address the problem of characterizing wealth based on a number of assets and household characteristics is conceptually appealing, but one might question whether the first principal component is all that is needed to capture the key aspects of wealth. Although Filmer and Pritchett clearly state that it is uncertain if the first principal component alone contains all the information relevant to a wealth index [11], they were presumably motivated by a desire to keep the method as simple as possible. Further, they encountered difficulties in explaining second and higher order components, even when they had relatively high variance [11,25]. After including higher order components in a multivariate regression analysis, they concluded that their results were robust to including additional components [8,11]. Nonetheless, some researchers have questioned this approach. Ward [26], while using only the first principal component in his analysis, notes that the second principal component appears to represent the rural structure of wealth and suggests that future research should look into the possibility of using higher-order principal components in the construction of wealth indices. Sharker et al. [21] also conclude that the first principal component may fail to incorporate correctly the contribution of all asset variables or to explain enough variability to ensure a correct classification of the subjects.

## Dummy variables in PCA

Yet another noteworthy aspect of the DHS method [27,28] is that categorical variables are converted into sets of binary (dummy) variables before performing the PCA. While it would usually be inappropriate to use the original codes of categorical variables – such as water source, type of toilet facility, materials for roof, walls and floor, etc. – directly in a PCA, it would nonetheless seem desirable to recode the categories sequentially from those involving fewer resources to those involving more. Filmer and Pritchett do not specifically explain in their original article [11] why they convert ordered categorical (ordinal) variables into binary variables. Presumably, this is to avoid making subjective judgements about the “wealth order” of the categories. However, using several binary variables to represent a single asset – an approach commonly used in regression analysis – is not as innocuous in PCA as it may appear. Kolenikov and Angeles [29,30] demonstrate that using (dummy) indicator variables in PCA construction of indices introduces spurious correlations if there are more than two categories for a variable, in addition to losing any ordinal information that applies. They propose instead the use of ordered categories, where they apply, and recommend the use of the polychoric [31-33] in place of the usual Pearson moment correlation matrix in the PCA. In this article, we will use the term “polychoric” generically to refer to tetrachoric (between two binary variables), polychoric (between two categorical variables) and polyserial (between a categorical and a continuous variable) correlations.

While the approach proposed by Kolenikov and Angeles has been used by some analysts [26,34,35], it has not been widely applied so far. Poirier et al. [9] reviewed studies which compare the performance of a polychoric PCA against the usual DHS index approach. They find that, while the two approaches are generally similar when compared to consumption and income measures, they diverge at the lower end of the SES spectrum. They conclude that there is a strong case to be made for the superiority of the polychoric PCA given that it overcomes the challenges relating to variable types and overcomes issues of “clumping” through greater discriminatory power at the lower end of the SES spectrum in the first principal component.

## Urban bias

Finally, the DHS index is known to be biased in favour of patterns of urban wealth: the so-called “urban bias” [19,36]. Because wealth tends to be concentrated in cities, the orientation of the axis of the first principal component is strongly influenced by aspects of wealth that are typically urban. Predominantly rural patterns of wealth, such as the ownership of agricultural land and domestic animals, tend to be poorly expressed along that axis. In fact, it is not uncommon to see negative coefficients for such items which, if used in the calculations, would effectively lower the wealth scores of households owning such assets. This would be equivalent to asserting, for example, that the more goats a farmer owns, the poorer s/he is, which clearly makes no sense. The methodology used to construct the DHS wealth index has benefitted from several developments over the years in an effort to improve its performance [28]. However, as will be shown in this article, the problem of the urban bias is still very prominent and justifies further research to address it effectively.

Adequately capturing both urban and rural patterns of wealth is of considerable importance as the DHS index is very widely used in equity analyses [37] and for policy making. For example, the World Bank used the index in a detailed analysis of socio-economic differences in health, nutrition and population in 56 developing countries [38], and many other studies have been published making use of this index. The validity of the conclusions drawn from such studies is placed in question if the measure of wealth on which they are based is systematically biased.

## METHODS

In this paper, we propose a new asset-based wealth index using: a) a similar set of assets as for the typical DHS index but in which categorical variables have been converted into ordered categorical (ordinal) variables, to harness additional discriminatory power and avoid the questionable use of multiple binary (dummy) variables to represent a single asset; b) squared multiple correlations (SMC) to remove variables weakly correlated with asset-based wealth and thus reduce “noise” in the PCA; c) a polychoric PCA, as methodologically more sound than a standard PCA for this application; and, most importantly, d) two principal components, instead of one, to take better account of key characteristics of rural wealth and redress the urban bias. We call this index the “polychoric dual-component wealth index” − P2C for short.

First, in a detailed example, we apply the P2C index on the Mozambique 2008 MICS – a survey in which two of the authors were closely involved and which motivated this research. We then present P2C results for 8 additional surveys and compare them with those of the DHS index and, in the case of Mozambique, with total expenditure. We end with a discussion on the merits of the new approach and potential policy implications. All statistical calculations presented in this paper were performed using STATA [39]. A STATA routine to calculate the P2C index is provided with this paper (P2C routine in the **Online Supplementary Document**).

### Conversion of categorical into ordered categorical variables

As already mentioned in the Introduction, Kolenikov and Angeles [29,30] have shown that the conversion of categorical into indicator variables, as done in the DHS index, introduces spurious correlations and, consequently, artificially modifies the orientation of the axes of the principal components. While it can be argued that ordering the categories of such variables as type of roof or toilet facilities introduces an element of subjectivity, in many instances this is minimal. For example, the relative cost/value of different types of floor, roof and wall materials, is usually well known. Furthermore, categories that are difficult to tell apart (in terms of comparative value) can be kept together in a joint category without invalidating the exercise. Regrouping categories thought to be of similar “value” is, in any case, regularly done during the calculation of any asset-based wealth index. For example, walls made of cane, palm or trunk were regrouped into a single answer category in the Mozambican MICS questionnaire, as is regularly done in typical surveys, on the assumption that they represent similar kinds of investments. Ownership of a car or a truck (obviously from a wide range of market prices) was also recorded in a single answer category in that questionnaire and there are numerous other such examples. Consequently, the proposition being made here, to classify various forms of a given asset into ordered categories, is a simple and logical extension of the method already in use in typical surveys.

### Squared multiple correlations

The PCA approach to calculating a wealth index is based on the assumption that each variable used in the analysis contributes information towards the asset-based wealth construct we are trying to measure. This is important because there is nothing other than the correlation (or shared variation [22]) between the variables to make this a wealth-oriented PCA. Variables that are not at all or only weakly related to asset-based wealth may add noise to the model and shift the axes of the principal components. It is best to leave such variables out of the analysis. The DHS and MICS do not use a fixed set of assets to calculate the wealth index, even though a core list is used as a starting point. Those who plan these surveys are urged to enlarge the basic list with assets they (subjectively) believe might help characterize wealth (or poverty) in their specific context. In practice, analysts calculating the DHS index commonly remove from the PCA variables that seem to perform counter-intuitively or appear to contribute little to the first principal component in order to achieve a satisfactory value (such as 10% or more) of its explained variance. This step relies largely on the judgement and experience of the analyst [27].

In an effort to reduce the arbitrariness of this process and make our method more replicable, we use squared multiple correlations (SMC) to screen variables for exclusion from the PCA as proposed by some analysts [23]. This is equivalent to calculating R^2^ in a regression of each variable on all the others. There is no established cut-off point below which a variable can be marked out as a weak contributor to a PCA. In this paper, we arbitrarily decided to use the value 0.05 as cut-off, ie, to exclude variables for which less than 5% of the variation can be explained by all the others jointly. It should be noted that once some of the variables have been removed a new SMC run must be performed in order to verify the squared multiple correlations of the smaller set of variables.

### Polychoric PCA

The reader is referred to the Introduction of this paper for the methodological justification for this approach in the literature. We provide below for the interested reader some technical details on the polychoric PCA as used here.

In the context of calculating a wealth index, the ordered categories of roofing material for example (from cheapest to most expensive) can be viewed as a discrete manifestation of an underlying (unobserved) continuous variable representing the “value” of the roof. The (standard) Pearson correlation coefficient tends to underestimate the strength of correlations in such cases while, conditional on some distributional assumptions, the polychoric correlation coefficient generally performs better. A simple example demonstrates this. Using any statistical software, generate 100 000 random values from a normal distribution with mean zero and unit standard deviation, and call this variable *X*. Let *Y* = *X* + 1. The variables *X* and *Y* are, by construction, perfectly correlated and Pearson correlation coefficient for *X* and *Y* is “1”. Let *X’* take the value “1” if *X* > 0 and “0” otherwise, and do similarly for *Y*. Pearson correlation coefficient for *X’* and *Y’* is now only around 0.4, suggesting a rather weak correlation between the two variables. The polychoric (or tetrachoric in this case) correlation coefficient for *X’* and *Y’*, however, is still “1”, reflecting the perfect correlation that exists between the two underlying continuous variables. Because PCA results depend essentially on the correlation matrix of the selected variables, the type of correlation used can have a marked effect on the results, as will later be demonstrated.

In the usual approach to the calculation of the DHS wealth index, the PCA is based on the correlation matrix of the variables included in the analysis, each categorical variable having previously been converted into a set of binary indicators. From a set of indicators X = [x_1_, x_2_, …, x_p_] a *p* × *p* sample correlation matrix C is derived. Each element C*_j,k_* of this matrix is estimated as the Pearson product moment correlation:


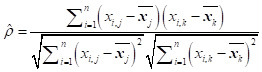


In the approach proposed in this paper, however, categorical variables are converted into ordinal (not dummy) variables. This results in a smaller *p’* × *p’* correlation matrix C’. For individual elements C’*_j,k_* of this matrix, the Pearson moment correlation coefficient is estimated only in the case of two continuous variables. When *x_j_* and *x_k_* are binary variables, we think instead of their correlation (ρ) as that between two latent bivariate normal distributed variables x*_j_ and x*_k_ giving rise to them:


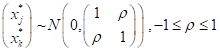


This can be estimated through any standard bivariate probit regression routine [40]. When *x_j_* and *x_k_* are ordinal variables with three or more categories (or a mix of binary and ordinal variables), a bivariate ordered probit routine is used to estimate ρ. In practice, the proportion of answers in each binary/ordered category is used to estimate, through a probit transformation, one or more thresholds over the latent continuous variable each binary/categorical variable is conceived to represent. The correlation coefficient between each pair of latent variables is then estimated by maximizing the likelihood function conditional on the threshold estimates. When *x_j_* is binary or ordinal and *x_k_* is continuous (or vice versa), a special routine implements a modification of the above approach in which the variance of the continuous variable is estimated directly from the sample while the binary/ordinal variable is viewed as deriving from a latent standard normal distribution. We end up with a hybrid correlation matrix obtained by combining the pairwise estimates of the polychoric, polyserial and moment correlations, on which the PCA is implemented in the usual manner by finding the eigenvectors for the estimated correlation matrix. In this analysis, we use the STATA command *polychoricpca* developed by Kolenikov and Angeles.

In terms of computational time, we ran the *polychoricpca* procedure on the Mozambican data set in 3.8 minutes using STATA version 16 on a 64-bit computer equipped with an Intel^®^ Core i-5 2.30 GHz CPU. While this is longer than the time used by the standard “pca” command, which runs in less than one second, it seems nonetheless reasonable for common analytical purposes.

### Two principal components

The final innovation of the approach we propose is the use of two principal components. It is worth once again emphasizing that using a single principal component, as done for the DHS index, is very unusual in PCA analyses. The goal of PCA is normally to identify the smallest possible set of principal components that captures an adequate proportion of total variation, and it is seldom the case that the 1st principal component, on its own, achieves that goal. Various methods have been proposed to identify the most meaningful set of principal components, including scree plots, which plot the variance accounted for by each principal component from the largest to the smallest. Scree plots are often used in PCA to determine by visual inspection which principal components contribute importantly to the analysis and should be retained. This is done by identifying the point on the graph where the decline in eigenvalues switches from roughly exponential to roughly linear [22,41].

Noting from our own observations and the literature [26] that the 2nd principal component in an asset-based polychoric PCA seems to express critical aspects of rural wealth, which would be potentially useful in redressing the urban bias suffered by the DHS index, we developed an approach to incorporate the first two principal components in the P2C index. We have not found elsewhere in the literature direct references to such an approach, except with respect to general methods of rotation in PCA. It is thus important to describe it in some detail.

The score vectors of a PCA – ie, the z-scores, following the terminology used Jackson [42] – are orthogonal (at 90°) to each other and uncorrelated. This is exactly true only for a standard PCA. In the case of a polychoric PCA, however, because the correlation matrix is based on the latent (continuous) variables that the binary and categorical asset variables are conceived to be derived from, the resulting score vectors are not exactly orthogonal, since they are estimated on the asset categories and not on the (unobserved) latent variables, as they would have to be if we were to obtain strictly orthogonal score vectors. Nonetheless, the principles are the same and the suggested approach remains a rational way to obtain the combined scores, as it would be in the case of a standard PCA.

If we project the scores of the first two principal components on a line 45° from their respective axes, we get a vector combining information proportionately from the two principal components; we call this new vector the “combined vector”. The amount of “information” in a PCA depends on the proportion of the total variance captured by each component, and the first component being always the one with the largest variance, the larger the proportion of the total variance captured by the 1st principal component in relation to the 2nd, the larger will be its contribution to the scores of the combined vector, which is exactly what we wish to have.

The operation described above corresponds to a rotation and is achieved by multiplying the matrix of the score vectors of the first two principal components by the following orthogonal rotation matrix:


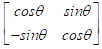


where *θ* is the clockwise angle of rotation of the axes, here 315° (45° counterclockwise) [22].

Rotations in PCA and factor analysis have been abundantly discussed in textbooks and literature [42]. In this case, since we retain only one axis (running through the 1st and 3rd quadrants), we effectively use only the 1st column vector of the above-mentioned rotation matrix and the operation reduces to:


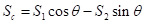


where

*S_c_* the combined score, and *S_1_* and *S_2_* are the scores of the 1^st^ and 2nd principal components.

As *θ* is 315° as previously indicated, the above formula reduces to:


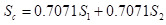


which is a simple addition of the scaled scores of the first two principal components. Because the scaling factor (0.7071) is identical for the two components, and because in a wealth index application we are essentially interested in the relative order of the scores rather than their absolute value, a simple addition of the unaltered first two principal component scores achieves the very same objective.

**Figure 1:**
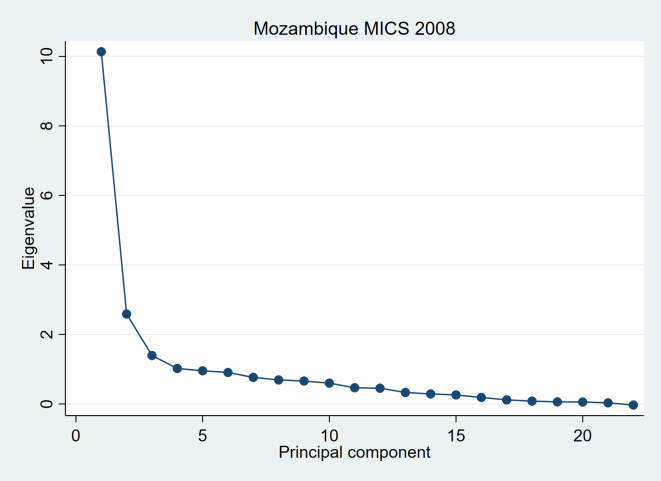
Scree plot of eigenvalues after a polychoric PCA, Mozambique MICS 2008.

An example of the above described method is presented below, using the Mozambique 2008 MICS data set. **Figure 1** presents the scree plot from a polychoric PCA on the asset variables later presented in this article. We see that the curve flattens from the 3rd principal component onward. This suggests that the first 2 principal components capture the substance of the shared variation between the asset variables. The remaining 20 principal components can be regarded as “noise”. Over a total variance of 22, the eigenvalue of the 1st principal component (pc1) is 10.1 and that of pc2 2.6. Thus, the first 2 principal components capture (explain) 58% of the total variance – or total information – in this PCA. This justifies using the first 2 principal components in the analysis.

**Figure 2:**
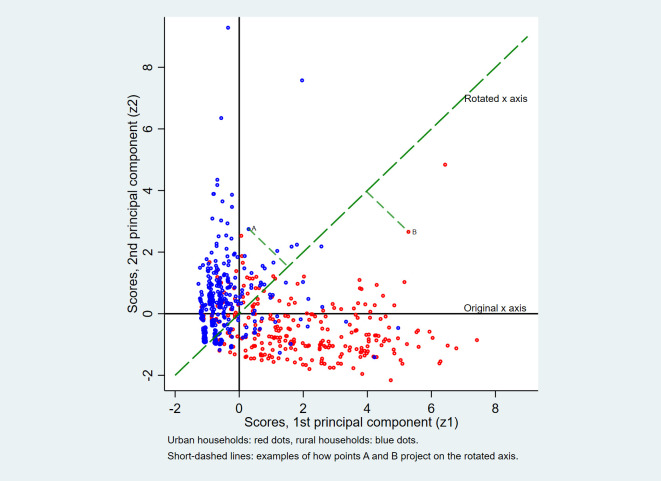
Orthogonal counterclockwise 45° axis rotation in PCA, Mozambique MICS 2008 data set (sub-sample*). *Sub-sample: the first household of each one of the 715 clusters of the survey.

**Figure 2** offers a graphic presentation of the method used to combine the scores of the first two principal components (x and y axes) along a single rotated axis (long-dashed line). A sub-sample of the data was used in order not to overload the graph. Two examples are shown (short-dashed lines) on how the data points project perpendicularly on the rotated axis. **Table 1** presents the numbers related to this method for the first and last 10 observations of the Mozambican data set. The table demonstrates that the ranking of observations based on a simple addition of the original pc1 and pc2 scores is identical to that based on the combined scores on the rotated x-axis. It can be demonstrated that the same procedure can be validly applied to combine 3 or more principal components if desired. Thus, if in a particular case the first two principal components were judged to capture too small a proportion of the total variance, it would be straightforward to add the third principal component if desired.

**Table 1:** Scores of 1st and 2nd principal components (original and rotated) and ranking, Mozambique MICS 2008 data set

Observations	z1: z-scores for pc1	z2: z-scores for pc2	z1r: z-scores for rotated pc1	z2r: z-scores for rotated pc2	ranking on z1r	z1 + 2: simple addition of z1 and z2	ranking on z1 + 2
1	-0.60	0.28	-0.22	0.62	6248	-0.32	6248
2	1.66	-0.05	1.13	-1.21	10 934	1.60	10 934
3	0.04	-0.06	-0.01	-0.06	7438	-0.02	7438
4	0.95	-1.20	-0.17	-1.52	6488	-0.25	6488
5	3.18	-0.41	1.96	-2.54	12 317	2.77	12 317
6	0.37	0.09	0.33	-0.20	8742	0.46	8742
7	1.12	-0.11	0.72	-0.87	9994	1.01	9994
8	0.73	-0.66	0.05	-0.98	7686	0.07	7686
9	-0.37	-0.54	-0.64	-0.12	4388	-0.91	4388
10	3.72	-0.74	2.11	-3.15	12 502	2.98	12 502
…							
13,946	2.92	-0.91	1.43	-2.71	11 458	2.02	11 458
13,947	2.83	-0.89	1.37	-2.63	11 400	1.94	11 400
13,948	2.83	-0.89	1.37	-2.63	11 401	1.94	11 401
13,949	3.66	-0.36	2.33	-2.84	12 738	3.30	12 738
13,950	3.96	0.09	2.86	-2.74	13 230	4.05	13 230
13,951	3.07	0.32	2.40	-1.94	12 802	3.39	12 802
13,952	-0.29	-1.25	-1.08	-0.68	2448	-1.53	2448
13,953	3.43	-0.96	1.75	-3.11	12 023	2.47	12 023
13,954	-0.23	-1.08	-0.93	-0.60	3075	-1.31	3075
13,955	-0.26	-0.88	-0.81	-0.44	3478	-1.14	3478

MICS – Multiple Indicator Cluster Survey, DHS – Demographic and Health Surveys, n – sample size, pc1 – principal component 1, etc

### Choice of data sets

The main approach used in this paper to investigate the urban bias is to look at the distribution of the wealth quintiles in large urban centres. Countries use different criteria to classify localities as being urban or rural; often, a population size criterion is used. However, populations classified as “urban” often contain a mix of urban and sub-urban populations, and sometimes a non-negligible proportion of typically rural populations practicing agriculture and animal husbandry. One of the authors remembers standing in the middle of a large corn field during a survey and wondering on what basis the Office of Statistics had classified that enumeration area as urban. It becomes difficult to demonstrate the urban bias when the so-called urban population is so heterogenous. Large cities, however, tend to comprise populations that are more uniformly urban in character. We also know that poverty is prevalent in practically all large urban centres, particularly in low- and middle-income countries. We presume that there is a strong urban bias when we find an almost complete absence of populations assigned to the lower wealth quintiles in large urban centres; conversely, we presume that the urban bias is negligible when urban poor (urban populations assigned to the lowest wealth quintiles) are in proportions more consistent with economic indicators and common sense. For this reason, the availability of data on at least one large urban centre, as a domain of analysis built into the design of the survey, was one of the criteria for the selection of the data sets used in this paper. Further, because an important objective of this paper is to demonstrate that the DHS wealth index continues to be subject to a substantial urban bias even after several methodological changes through its history [28], and to demonstrate that the P2C index addresses such bias, we selected surveys over a sufficiently long time period in order to include DHS wealth indices based on the older (single PCA) and more recent (composite: combining urban and rural PCAs) DHS approaches [27,28].

## RESULTS

### A detailed example: the Mozambique 2008 MICS

**Table 2** shows the estimated distribution of the Mozambican population according to standard (DHS) wealth quintiles as compiled for the MICS 2008 [43]; this wealth index was calculated from a single PCA as was the standard approach at the time. In Maputo City, we note a complete absence of individuals in the lowest three quintiles, and a mere 4% in the 4th quintile, leaving 96% of the capital’s population in the highest (wealthiest) quintile; this is a typical example of urban bias and it motivated the authors to undertake this research. Similar patterns for Maputo City are observed in the Mozambican DHS 2003[44], DHS 1997 [45] and DHS 2011 [46] (data not shown).

**Table 2:** Distribution of the population by DHS wealth quintiles — Mozambique MICS 2008

Sub-population	n	DHS wealth quintile (individuals)				
**1^st^ (poorest)**	**2^nd^**	**3^rd^**	**4^th^**	**5^th^ (wealthiest)**
Overall	65 524	20.0%	20.0%	20.0%	20.0%	20.0%
Urban	29 403	4.2%	6.6%	11.4%	24.7%	53.0%
Rural	36 121	27.7%	26.4%	24.2%	17.7%	4.0%
Niassa	5176	20.2%	27.3%	30.8%	14.9%	6.9%
Cabo Delgado	5156	17.0%	32.5%	30.0%	15.6%	5.0%
Nampula	6421	26.3%	22.5%	23.8%	15.9%	11.5%
Zambézia	6577	42.9%	24.6%	17.1%	11.8%	3.6%
Tete	5313	24.3%	29.3%	27.3%	14.8%	4.3%
Manica	5725	18.1%	23.5%	28.5%	18.1%	11.8%
Sofala	7394	16.9%	20.4%	18.6%	18.7%	25.4%
Inhambane	5258	5.5%	11.0%	19.8%	45.7%	17.9%
Gaza	5943	1.7%	4.5%	12.0%	52.9%	28.9%
Maputo Prov.	5451	0.6%	2.1%	4.3%	28.3%	64.6%
Maputo City	7110	0.0%	0.0%	0.0%	4.2%	95.8%

MICS – Multiple Indicator Cluster Survey, DHS – Demographic and Health Surveys, n – sample size

**Table 3** lists 60 variables from the Mozambican MICS 2008 potentially relevant to a wealth index oriented PCA analysis. As is typically done with the DHS wealth index, categorical variables (eg, roof material) have been converted into a set of binary variables each expressing a single category. We ran a PCA on these variables. As shown in the left portion of the table, the 1st component captures only 15% of the total variance, the 2nd 6%, the 3rd 4%, etc. (only the results for the first 3 principal components are shown here in detail for economy of space). Examining the eigenvectors of the 1st principal component, we observe that the more strongly positive coefficients tend to relate to assets characteristic of wealth (eg, cement floor, wall made of cement blocks, ownership of a television or a mobile phone, etc.). The more negative coefficients tend to relate to assets characteristic of poverty (earth floor, roof made of thatch or palm leaves, using wood as fuel for cooking, etc.). However, there are some odd results as well. For example, the ownership of a bicycle has a negative effect on the wealth index score; the coefficients for parquet and ceramic floors are lower than those for bare cement. While ownership of cattle carries a positive coefficient (albeit a weak one), the coefficients for goats, sheep, pigs and chicken are all negative; this is equivalent to saying that the more sheep a farmer owns, the poorer s/he is, which clearly makes no sense. Removing agricultural land and domestic animals from the analysis, as was usually done in calculating the original DHS wealth index (with a single PCA), doesn’t markedly affect the other coefficients, and barely increases the proportion of the total variance captured by the first principal component (data not shown).

**Table 3:** DHS PCA on binary variables vs standard PCA on ordered categorical variables − Mozambique MICS 2008

DHS approach†				Standard PCA on ordered categorical variables			
**Components**	**Eigenvalue**	**Prop. of variance**	**Cumul. prop.**	**Components**	**Eigenvalue**	**Prop. of variance**	**Cumul. prop.**
Comp. 1	8.79	0.15	0.15	Comp. 1	6.48	0.25	0.25
Comp. 2	3.42	0.06	0.20	Comp. 2	2.14	0.08	0.33
Comp. 3	2.29	0.04	0.24	Comp. 3	1.40	0.05	0.39
Comp. 4	1.90	0.03	0.27	Comp. 4	1.17	0.05	0.43
Comp. 5	1.80	0.03	0.30	Comp. 5	1.09	0.04	0.47
**Variables (binary)**	**Eigenvectors**			**Variables (ordered categories)**	**Eigenvectors**		
	**Comp. 1**	**Comp. 2**	**Comp. 3**		**Comp. 1**	**Comp. 2**	**Comp. 3**
Persons/sleeping room*	-0.045	0.000	0.123	Persons/sleeping room*	-0.051	0.124	-0.170
Floor: earth/sand	-0.174	0.055	-0.203	Floor	0.319	-0.033	0.083
Floor: dung	-0.079	0.043	0.238	…			
Floor: rough wood	0.005	0.012	0.003	…			
Floor: cement	0.249	-0.222	0.017	…			
Floor: parquet/fine wood	0.101	0.333	-0.050	…			
Floor: ceramic tiles	0.085	0.164	0.001	…			
Roof: thatch/palm leaves	-0.267	0.145	-0.028	Roof	0.222	-0.027	0.030
Roof: metal	0.206	-0.264	0.048	…			
Roof: calamine/fib.cement	0.093	0.016	-0.010	…			
Roof: ceramic tiles	0.035	0.033	0.008	…			
Roof: cement	0.130	0.365	-0.054	…			
Walls: cane/palm/trunk	-0.016	-0.075	-0.054	Walls	0.302	-0.037	0.094
Walls: bamboo & mud	-0.101	0.044	-0.083	…			
Walls: uncovered adobe	-0.108	0.050	0.133	…			
Walls: wood/zinc	0.026	-0.055	0.005	…			
Walls: cement blocks/tiles	0.267	-0.030	-0.011	…			
Fuel: wood	-0.265	0.053	0.115	Fuel	0.313	-0.101	-0.006
Fuel: animal dung	-0.005	0.002	-0.036	…			
Fuel: kerosene	0.005	-0.014	-0.025	…			
Fuel: coal	0.010	-0.008	-0.010	…			
Fuel: charcoal	0.215	-0.185	-0.098	…			
Fuel: LPG	0.145	0.291	-0.038	…			
Fuel: electricity	0.066	0.074	-0.005	…			
Electricity	0.273	0.028	-0.022	Electricity	0.325	-0.042	-0.031
Radio	0.097	0.021	0.270	Radio	0.125	0.283	-0.407
Television	0.271	0.023	0.019	Television:	0.323	0.010	-0.034
Mobile phone	0.255	-0.076	0.061	Mobile phone	0.287	0.043	-0.020
Non-mobile phone	0.087	0.162	0.006	Non-mobile phone	0.116	0.022	0.020
Refrigerator	0.248	0.140	0.010	Refrigerator	0.310	0.005	0.002
Watch	0.080	0.054	0.225	Watch	0.108	0.244	-0.359
Bicycle	-0.058	0.054	0.311	Bicycle	-0.051	0.327	-0.442
Motorcycle/scooter	0.063	-0.016	0.159	Motorcycle/scooter	0.082	0.155	-0.133
Animal drawn cart	0.013	-0.006	0.261	Animal drawn cart	0.017	0.305	0.333
Car/truck	0.142	0.219	0.061	Car/truck	0.191	0.083	0.080
Boat with motor	0.016	0.011	0.036	Boat with motor	0.020	0.045	0.140
Agric. land (hectares)*	-0.025	0.042	0.158	Agric. land (hectares)*	-0.024	0.170	0.006
No. of cattle*	0.013	0.015	0.303	No. of cattle*	0.018	0.372	0.427
No. of goats*	-0.006	0.026	0.336	No. of goats*	-0.002	0.401	0.228
No. of sheep*	-0.002	0.019	0.202	No. of sheep*	0.001	0.228	0.238
No. of pigs*	-0.011	0.005	0.236	No. of pigs*	-0.013	0.260	0.061
No. of chicken*	-0.025	0.036	0.281	No. of chicken*	-0.021	0.347	-0.087
No. of ducks*	0.044	-0.034	0.130	No. of ducks*	0.049	0.159	-0.064
Water: surface	-0.076	0.051	0.067	Water	0.274	-0.036	0.021
Water: public tap	0.060	-0.111	-0.050	…			
Water: neighbour's house	0.108	-0.154	-0.097	…			
Water: unprotected well	-0.118	0.056	-0.056	…			
Water: prot. well − pump	0.018	-0.049	0.017	…			
Water: prot. well + pump	-0.048	-0.010	0.106	…			
Water: rainwater	0.008	-0.025	0.028	…			
Water: piped in yard/plot	0.162	-0.015	-0.020	…			
Water: piped into dwelling	0.138	0.255	-0.019	…			
Water: bottled	0.039	0.147	-0.019	…			
Toilet: bush/field/none	-0.171	0.110	-0.124	Toilet	0.321	-0.030	-0.007
Toilet: pit latrine − slab	-0.014	-0.102	0.158	…			
Toilet: pit latrine + slab	0.080	-0.110	0.016	…			
Toilet: VIP latrine	0.129	-0.142	-0.046	…			
Toilet: flush to septic tank	0.161	0.077	0.000	…			
Toilet: flush to sewer	0.118	0.368	-0.058	…			
Toilet: flush to unknown	0.004	0.007	0.002	…			

MICS – Multiple Indicator Cluster Survey, DHS – Demographic and Health Surveys, PCA – Principal Component Analysis, comp. 1: principal component 1, etc

*Continuous or count variables.

†Recalculated.

Examining the 2nd principal component, it is not easy to make sense of the pattern of wealth it is expressing, except that it seems to accentuate some of the very urban and “upper class” characteristics—eg, parquet or ceramic floor, ownership of a car, in-house running water—in contrast to more “popular class” characteristics of urban wealth. The interpretation of the 3rd principal component is, however, unmistakable, as it loads heavily on rural characteristics of wealth, eg, ownership of an animal drawn cart, of a bicycle, the number of domestic animals owned, etc.

Having examined the performance of a PCA using the typical DHS approach, we now examine how a standard PCA performs using ordered categories instead of binary variables, where applicable. The results for the Mozambican data set are presented in the right portion of **Table 3**. We now have 26 variables instead of 60. As expected, reducing the number of variables results in an increase in the proportion of the total variance captured by the 1st and 2nd principal components (25% and 8% of the total variance respectively). The 1st component continues to express poorly typically rural characteristics of wealth with, as before, negative coefficients for some domestic animals. Of note, however, the 2nd principal component now features strong rural characteristics, as was observed for the 3rd component of the PCA of the original DHS approach.

**Figure 3:**
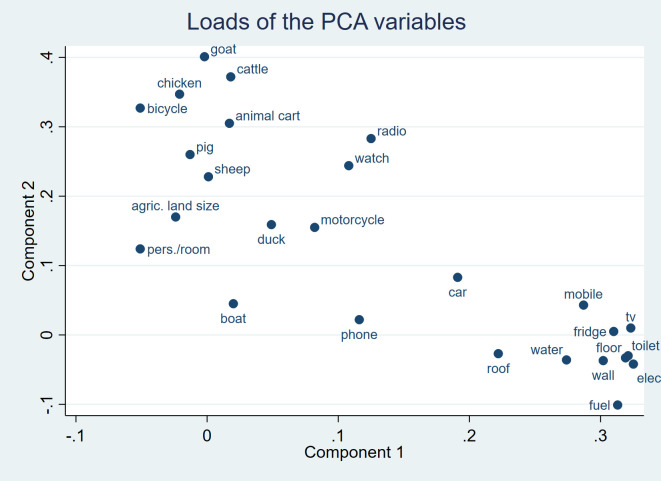
Distribution of coefficients along principal components 1 and 2 of a standard PCA on ordered categorical variables − Mozambique MICS 2008.

Continuing with ordinal variables, **Figure 3** shows a scatter plot of the coefficients for each variable in the first two principal components (sometimes referred to as “loads”). Bicycle, goat, cattle and chicken occupy the top left quadrant, making little contribution to the 1st, but strong contributions to the 2nd principal component. Watch, radio and motorcycle are found near the centre of the graph indicating that these wealth characteristics contribute fairly equally to the 1st and 2nd principal components. Variables such as type of cooking fuel, electricity, toilet type are found in the lower right quadrant, making a strong contribution to the 1st, but little contribution to the 2nd principal component.

The left portion of **Table 4** shows the results obtained when running a polychoric instead of a standard PCA with the previous set of ordered categorical (and a few continuous) variables. It is worth noting that, in a polychoric PCA, a coefficient is assigned to each category of an asset variable, while in a standard PCA there is a single coefficient per variable. This stems from the fact that, while a standard PCA operates on each variable as though it were continuous (even when it is not), a polychoric PCA considers that each variable category projects on a segment of the distribution of the latent (continuous) variable it is conceived to be derived from, each with its own coefficient.

**Table 4:** Polychoric PCA on a full and retained set of variables after SMC − Mozambique MICS 2008

	Polychoric PCA (full set of variables)			Polychoric PCA (retained variables)		
**Components**	**Eigen value**	**Prop. of variance**	**Cumul. prop.**	**Eigen value**	**Prop. of variance**	**Cumul. prop.**
Comp. 1	10.27	0.40	0.40	10.13	0.46	0.46
Comp. 2	2.71	0.10	0.50	2.59	0.12	0.58
Comp. 3	1.56	0.06	0.56	1.39	0.06	0.64
Comp. 4	1.38	0.05	0.61	1.02	0.05	0.69
Comp. 5	1.04	0.04	0.65	0.95	0.04	0.73
**Variables (ordered categories)**	**Eigenvectors**			**Eigenvectors**		
	**Comp. 1**	**Comp. 2**	**Comp. 3**	**Comp. 1**	**Comp. 2**	**Comp. 3**
Persons/sleeping room*	-0.055	0.131	-0.163	n/a	n/a	n/a
Floor 1: earth/sand/dung	-0.112	0.028	-0.024	-0.113	0.028	-0.048
Floor 2: rough wood	0.219	-0.054	0.047	0.220	-0.055	0.093
Floor 3: cement	0.360	-0.088	0.077	0.363	-0.090	0.153
Floor 4: parquet/tiles/etc.	0.722	-0.177	0.155	0.727	-0.180	0.307
Roof 1: thatch/palm/metal	-0.024	0.007	0.005	-0.024	0.007	-0.001
Roof 2: calamine/fibre cement	0.487	-0.151	-0.101	0.492	-0.144	0.027
Roof 3: cement/tiles	0.631	-0.196	-0.132	0.638	-0.187	0.035
Walls 1: cane/palm/mud/adobe	-0.090	0.021	-0.042	-0.090	0.024	-0.042
Walls 2: wood/zinc	0.264	-0.063	0.124	0.264	-0.071	0.123
Walls 3: cement blocks/tiles	0.418	-0.100	0.197	0.419	-0.113	0.195
Fuel 1: wood/dung	-0.085	0.061	0.050	-0.086	0.059	0.019
Fuel 2: kerosene/coal/charcoal	0.364	-0.258	-0.215	0.369	-0.250	-0.082
Fuel 3: LPG/electricity	0.637	-0.452	-0.376	0.646	-0.437	-0.144
Electricity: no	-0.075	0.018	0.015	-0.075	0.018	0.006
Electricity: yes	0.475	-0.114	-0.096	0.479	-0.112	-0.041
Radio: no	-0.129	-0.272	0.257	-0.132	-0.295	0.206
Radio: yes	0.124	0.261	-0.247	0.126	0.282	-0.197
Television: no	-0.077	-0.001	0.014	-0.078	-0.002	0.003
Television: yes	0.472	0.006	-0.085	0.477	0.011	-0.017
Mobile phone: no	-0.119	-0.012	0.015	-0.120	-0.014	-0.017
Mobile phone: yes	0.357	0.036	-0.044	0.360	0.041	0.051
Non-mobile phone: no	-0.005	-0.001	-0.002	-0.005	-0.001	0.000
Non-mobile phone: yes	0.647	0.119	0.209	0.647	0.104	-0.027
Refrigerator: no	-0.049	0.001	0.002	-0.049	0.001	-0.002
Refrigerator: yes	0.563	-0.016	-0.025	0.566	-0.014	0.029
Watch: no	-0.056	-0.121	0.088	-0.057	-0.129	0.123
Watch: yes	0.167	0.362	-0.263	0.169	0.385	-0.367
Bicycle: no	0.033	-0.270	0.157	0.033	-0.283	0.226
Bicycle: yes	-0.050	0.404	-0.236	-0.050	0.424	-0.338
Motorcycle/scooter: no	-0.011	-0.019	0.000	-0.011	-0.020	0.016
Motorcycle/scooter: yes	0.322	0.587	0.006	0.322	0.596	-0.488
Animal drawn cart: no	-0.002	-0.013	-0.013	-0.002	-0.013	-0.001
Animal drawn cart: yes	0.129	0.974	0.971	0.119	0.955	0.109
Car/truck: no	-0.016	-0.006	-0.008	-0.016	-0.006	-0.003
Car/truck: yes	0.615	0.238	0.300	0.615	0.222	0.135
Boat with motor: no	-0.001	-0.001	-0.003	n/a	n/a	n/a
Boat with motor: yes	0.321	0.386	1.997	n/a	n/a	n/a
Agric. land (hectares)*	-0.025	0.152	0.005	n/a	n/a	n/a
No. of cattle*	0.014	0.227	0.209	0.014	0.233	0.491
No. of goats*	0.000	0.285	0.043	-0.001	0.297	0.412
No. of sheep*	0.002	0.143	0.126	0.001	0.150	0.397
No. of pigs*	-0.016	0.214	0.114	-0.017	0.218	0.242
No. of chicken*	-0.016	0.269	-0.238	-0.014	0.291	0.101
No. of ducks*	0.034	0.109	-0.161	n/a	n/a	n/a
Water 1: public/neighbour	-0.048	0.016	0.008	-0.048	0.016	-0.006
Water 2: piped in yard/plot	0.427	-0.143	-0.073	0.430	-0.140	0.050
Water 3: piped into dwelling	0.618	-0.207	-0.106	0.623	-0.203	0.072
Water 4: bottled	0.885	-0.296	-0.152	0.893	-0.291	0.103
Toilet 1: public/neighbour/field	-0.209	0.033	0.044	-0.211	0.033	-0.009
Toilet 2: pit latrine − slab	0.122	-0.019	-0.026	0.123	-0.019	0.005
Toilet 3: pit latrine + slab	0.298	-0.047	-0.063	0.302	-0.047	0.012
Toilet 4: VIP latrine	0.390	-0.061	-0.082	0.395	-0.061	0.016
Toilet 5: without flush	0.487	-0.077	-0.103	0.493	-0.076	0.020
Toilet 6: with flush	0.628	-0.099	-0.132	0.636	-0.098	0.026

*Continuous or count variables. n/a: not available.

MICS – Multiple Indicator Cluster Survey, PCA – Principal Component Analysis, prop. – proportion, cumul. – cumulative, comp. 1 – principal component 1, etc

The polychoric correlation approach results in a markedly larger proportion of the variance being explained by the first principal component (increased from 25% to 40%), although the number of variables is unchanged from the standard PCA analysis using ordinal variables. The second principal component shows a smaller increase in the proportion of the variance explained (from 8% to 10%), and the first two principal components now jointly capture 50% of the total variance. The 1st principal component still appears to reflect mainly urban, and the 2nd mainly rural, patterns of wealth. Further, for binary (yes/no) variables in this example, when considering the sum of the coefficients of the first two components, ownership is always associated with a positive overall value and non-ownership with a negative value. In the case of variables with more than two (ordered) categories, the sum of the coefficients of the first two components always increases when moving from a lower category to a higher one. For count variables (eg, number of animals owned), the sum of the coefficients of the first two components is always positive.

As explained in the Methods section of this paper, we use squared multiple correlations (SMC) to exclude variables for which less than 5% of the variation can be explained by all the others jointly, thus retaining in the PCA only variables that contribute meaningfully to characterising asset-based wealth and reducing noise. In the Mozambican data set, this procedure resulted in the exclusion of 4 variables: number of persons per room, boat ownership, agricultural land size and ownership of ducks. The results of a new polychoric PCA performed on the 22 retained variables are presented in the right section of **Table 4****.** The 1st principal component captures 46% of the total variance of the retained variables and the 2nd component 12%, resulting in 58% of the total variance being explained by these first two principal components. Judging by the size of the coefficients (eigenvectors) of the first two principal components, all 22 variables contribute importantly to at least one of the first two principal components. The characterization of the second principal component as expressing typically rural aspects of wealth remains valid. **Figure 1** shows a scree plot of the eigenvalues from which it is quite clear in this case that the third eigenvalue is where the transition of the curve from roughly exponential to roughly linear happens, indicating that in this analysis the first two components both express inherent characteristics of asset-based wealth, while the others may be regarded as noise.

We now calculate a wealth score integrating the first two principal components using the approached already described in the Methods section of this paper. As usual, wealth scores are first assigned to households and individuals are then allocated the score of their respective household. Transforming these new wealth scores into wealth quintiles in our Mozambican data set results in 55% of individuals previously classified as belonging to the poorest quintile according to the DHS index being reclassified into higher quintiles of wealth, and 30% formerly in the wealthiest quintile being reclassified into lower quintiles (data not shown). Most changes, however, occur in the 2nd, middle and 4th quintiles, with about three quarters of individuals being reclassified downward or upward. In the urban population 40% of individuals were “downgraded” while 8% were “upgraded”. In the rural population 40% of individuals were upgraded, many of them by two or more quintiles, while 25% were downgraded.

The distribution of the Mozambican population using these new wealth quintiles is shown in **Table 5**. The most striking difference between these results and those of the DHS quintiles, shown in **Table 2**, is with respect to Maputo City. The P2C index results in a more plausible distribution of the capital’s population across the wealth quintiles, each one of which now has a proportion of the population assigned to them. While only 0.5% of the capital’s population falls into the poorest quintile, the second quintile now contains 5% and the middle quintile 7%, where the DHS index previously showed no-one at all. The 4th quintile has increased from 4% to 21%, with only 67% of the capital’s population now classified as belonging to the wealthiest quintile, compared with 96% in the previous estimate. At the provincial level the results of the new approach look credible overall and are generally more evenly distributed across the quintiles. In particular, Inhambane and Gaza have more plausible looking distributions compared with the standard analysis which resulted in improbably large peaks in the fourth quintile. Maputo Province too now has more credible proportions of the population in the lower quintiles.

**Table 5:** Distribution of population by P2C wealth quintile — Mozambique MICS 2008

Sub-population	n	P2C wealth quintile (individuals)				
**1^st^ (poorest)**	**2^nd^**	**3^rd^**	**4^th^**	**5^th^ (wealthiest)**
Overall	65 524	20.1%	19.9%	20.0%	20.0%	20.0%
Urban	29 403	9.5%	13.5%	16.1%	22.5%	38.4%
Rural	36 121	25.3%	23.0%	21.9%	18.7%	11.1%
Niassa	5176	16.8%	22.4%	25.2%	25.7%	9.9%
Cabo Delgado	5156	25.9%	25.5%	26.3%	16.2%	6.2%
Nampula	6421	32.2%	23.2%	19.6%	12.4%	12.6%
Zambézia	6577	26.5%	24.0%	23.5%	20.2%	5.7%
Tete	5313	26.4%	21.4%	20.5%	18.1%	13.7%
Manica	5725	18.9%	19.5%	16.3%	26.1%	19.1%
Sofala	7394	10.1%	17.8%	20.9%	25.7%	25.4%
Inhambane	5258	14.0%	18.6%	19.3%	23.6%	24.5%
Gaza	5943	11.0%	14.6%	14.5%	23.2%	36.8%
Maputo Prov.	5451	6.1%	11.1%	15.7%	22.2%	44.9%
Maputo City	7110	0.5%	4.9%	7.2%	20.5%	66.9%

MICS – Multiple Indicator Cluster Survey, P2C – polychoric dual-component wealth index, n – sample size

**Figure 4:**
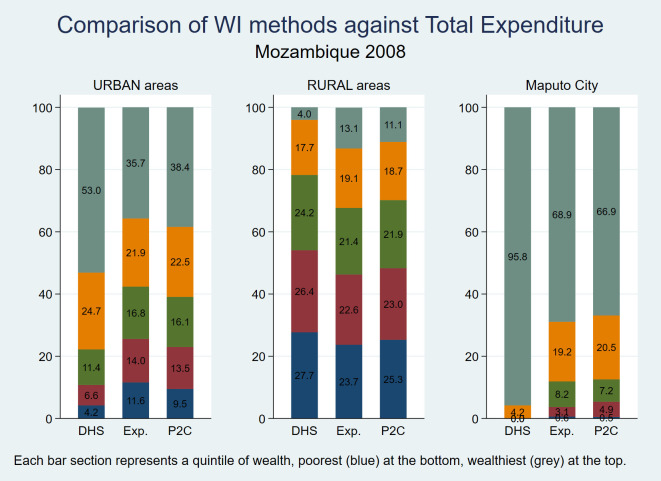
Distribution of population by DHS, P2C and per capita expenditure (Exp.) wealth quintiles − Mozambique 2008.

In an attempt to validate the proposed method, we compared the wealth quintile distributions derived using the DHS and P2C approaches with those based on per capita expenditure derived from the national household budget survey [47,48] conducted by the National Institute of Statistics of Mozambique in 2008/2009. While we do not expect a measure of wealth based on durable assets to be exactly equivalent to one based on current expenditure, it would be reassuring if the two were closely related. As shown in **Figure 4**, the distribution of the P2C wealth quintiles is clearly more consistent with that of the per capita expenditure measure than the DHS method, especially for the urban population, and for Maputo City in particular. For individual provinces, the per capita expenditure quintiles appear consistent with the method proposed in this paper (data not shown). Ideally, we would perform an individual-level comparison of each asset-based index against per capita expenditure, but suitable individual-level data required to calculate a standard wealth index to make such a comparison were not available in the household budget survey. Since a measure of individual agreement could not be estimated, we calculated a weighted χ^2^ statistic to compare the distribution of each of the asset-based indices with that of expenditure data. This is not an attempt to show if differences are statistically significant but rather to use the χ^2^ statistic as a form of population level measure of agreement between the distributions, large χ^2^ values suggesting large distributional differences and vice-versa. Comparing the distribution of the DHS index with that of the per capita expenditure, we obtain weighted (design-based) χ^2^ values of 18.74, 19.53 and 38.88 for the urban, rural and Maputo City distributions respectively – an expression of the often large differences apparent in **Figure 4****.** For the P2C index, those values are 0.96, 0.98 and 0.90 respectively, confirming that, at least in the case of Mozambique, the wealth quintile distribution arrived at through the method proposed in this paper correlates much better with expenditure data at sub-population level than does the DHS index.

The effects that such method-related differences in the assessment of socio-economic status have on equity analyses of various indicators will depend on how each indicator actually correlates with wealth. We present three examples in **Table 6****.** The upper section of the table refers to the percentage of women age 15-49 years giving birth in the last two years who were delivered by a skilled attendant. In Mozambique, such services are offered nearly free of charge at government hospitals and are thus easily accessed by both rich and poor in large urban centres, while access is lower for those in rural areas. Overall, the P2C index shows a relatively high proportion of skilled attendance among the urban poor, while the DHS approach suggests larger inter-quintile differences. With respect to the percentage of children weighed at birth, shown in the middle portion of the table, the differences between the two indices are even more accentuated. Thus, for these two access related indicators, the analysis based on the P2C index suggests less inequity than that based on the DHS index, as reflected in the concentration index values (based on the wealth scores) shown in the table. In the case of the percentage of women 15 to 49 years with secondary education in the lower portion of **Table 6** – an indicator also strongly related to access but likely to be more dependent on wealth than the two previous ones – while the overall distribution by wealth quintiles is comparable for the two approaches, the distribution by area is notably different, especially for the urban population, with the concentration index values pointing once again at less inequity when using the P2C approach.

**Table 6:** Results of three indicators by wealth quintile, area and concentration index − Mozambique MICS 2008

Women 15-49 age group	Wealth quintiles					All	Conc. index	n
**1^st^ (poorest)**	**2^nd^**	**3^rd^**	**4^th^**	**5^th^ (wealthiest)**
**Skilled attendant at last delivery**								
DHS index:								
Mozambique – Urban	40.5%	57.3%	69.3%	79.1%	90.6%	78.3%	0.101	1914
Mozambique – Rural	36.1%	43.5%	48.6%	57.1%	78.4%	45.9%	0.112	2990
Mozambique – Overall	36.5%	45.1%	52.6%	66.2%	88.5%	55.3%	0.175	4904
P2C index:								
Mozambique – Urban	62.9%	70.9%	76.4%	78.4%	90.3%	78.3%	0.067	1914
Mozambique – Rural	40.3%	44.1%	47.5%	47.7%	58.0%	45.9%	0.058	2990
Mozambique – Overall	44.0%	50.2%	54.3%	58.5%	75.5%	55.3%	0.099	4904
**Last child weighed at birth**								
DHS index:								
Mozambique – Urban	42.2%	66.1%	68.1%	84.2%	96.5%	83.0%	0.103	1914
Mozambique – Rural	39.6%	44.5%	50.4%	60.1%	81.3%	48.3%	0.102	2990
Mozambique – Overall	39.8%	47.1%	53.8%	70.0%	93.8%	58.3%	0.172	4904
P2C index:								
Mozambique – Urban	70.5%	74.6%	77.8%	84.6%	95.2%	83.0%	0.064	1914
Mozambique – Rural	43.2%	44.7%	49.5%	51.1%	61.7%	48.3%	0.062	2990
Mozambique – Overall	47.7%	51.5%	56.1%	62.9%	79.8%	58.3%	0.101	4904
**Secondary education**								
DHS index:								
Maputo City	n/a	n/a	n/a	13.3%	47.4%	46.2%	0.287	1948
Mozambique – Urban	1.4%	3.2%	9.0%	15.4%	45.9%	31.0%	0.406	6960
Mozambique – Rural	0.6%	1.4%	2.1%	9.0%	23.9%	3.7%	0.606	7228
Mozambique – Overall	0.6%	1.6%	3.4%	11.7%	43.2%	13.6%	0.662	14188
P2C index:								
Maputo City	(*)	15.6%	19.9%	27.6%	57.1%	46.2%	0.267	1948
Mozambique – Urban	6.4%	10.6%	15.3%	24.0%	52.6%	31.0%	0.375	6960
Mozambique – Rural	1.2%	2.2%	3.0%	5.1%	11.5%	3.7%	0.421	7228
Mozambique – Overall	2.1%	4.2%	6.4%	12.8%	39.1%	13.6%	0.545	14188

n/a – not available, MICS – Multiple Indicator Cluster Survey, DHS – Demographic and Health Surveys. P2C – polychoric dual-component wealth index, conc. – concentration, n – sample size

*Result suppressed due to small sample size. Results for Maputo City not shown for first two indicators due to small sample size in lower quintiles.

### Additional results

To examine further the P2C approach, data sets from four countries, for which a MICS data set from the same round as that of the previously analysed Mozambican data set was available – using the original DHS method with a single PCA – and for which economic data published by the World Bank [49] for the same survey year were available as well, were analysed. These are Ghana 2006 [50], Vietnam 2006 [51], Mongolia 2005 [52] and Albania 2005 [53] (Tables S1 and S2 in the **Online Supplementary Document**). All four scree plots support the inclusion of the 2nd principal component in the index except perhaps that for Vietnam, where evidence for the need for the 2nd component is weaker. For all four P2C PCAs, over 50% of the variation expressed by the variables is captured by the first two principal components: 56% for Ghana, 52% for Vietnam, 60% for Mongolia and 57% for Albania (data not shown). Furthermore, in all four countries, the P2C method produces higher, more plausible, proportions of urban poor and rural wealthy than the DHS approach (Table S2 in the **Online Supplementary Document**). This is noteworthy as the countries analysed here have rather different profiles, including one (Albania) with much lower levels of absolute poverty than the others. In the years of the surveys, Albania had less than 8% of its population living on less than US$2/d, while the other three countries all had about 50%.

**Figure 5:**
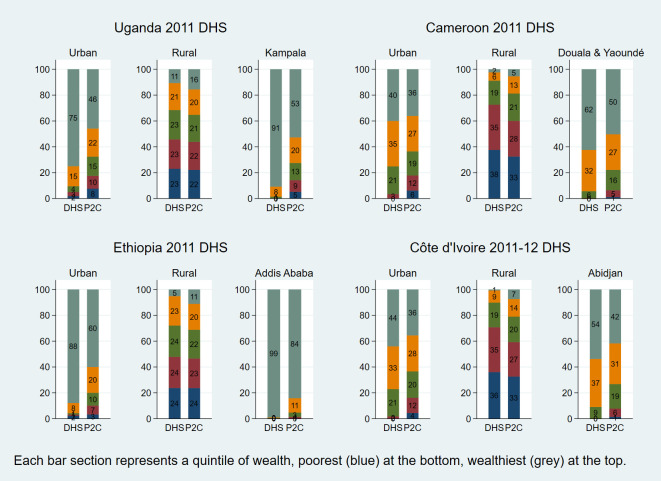
Distribution of population by new (composite) DHS and P2C wealth quintile −4 countries.

As previously mentioned, DHS has in recent years introduced a new composite wealth index which uses three PCAs instead of one, in an effort to improve the performance of the index and reduce the urban bias. Results from this newer (composite) approach have been published in DHS reports from 2011 onward. We examined four DHS surveys implemented on the African continent that have used this composite DHS wealth index and which include results for large urban centres allowing us to assess the urban bias. We compared the published results with those of the P2C index proposed in this paper. These are Cameroon 2011 [54], Ethiopia 2011 [55], Uganda 2011 [56] and Côte d’Ivoire 2011-12 [57]. **Figure 5** compares the distributions of the new (composite) DHS and P2C wealth quintiles for these four countries. These are in many ways comparable to **Figure 4** as far as the two indices are concerned and clearly indicate that the urban bias still persists under the composite DHS approach, with implausibly low proportions of individuals in the lowest quintiles in large urban centres, as was the case with the original DHS index using a single PCA. The P2C approach continues to give more credible estimates of the proportions of urban poor.

**Table 7** summarizes the distribution by wealth quintiles of the populations of six large, strictly urban areas extracted from the eight above-mentioned data sets: Greater Accra (urban) in Ghana, Ulaanbaatar in Mongolia, Douala/Yaoundé in Cameroon, Abidjan in Côte d’Ivoire, Addis Ababa in Ethiopia and Kampala in Uganda. The P2C approach generally identifies more credible proportions of urban poor in those large urban areas than the DHS method including the four cases where the new composite DHS index method was used (marked with an asterisks).

**Table 7:** Distribution of population by DHS and P2C wealth quintiles — 6 large urban areas

Sub-population	n	Wealth quintile (individuals)				
**1^st^ (poorest)**	**2^nd^**	**3^rd^**	**4^th^**	**5^th^ (wealthiest)**
**DHS wealth quintiles**						
Greater Accra (urban), Ghana 2006	2795	0.0%	0.0%	6.1%	26.8%	67.1%
Ulaanbaatar, Mongolia 2005	9084	0.4%	6.5%	22.2%	34.5%	36.3%
Douala/Yaoundé, Cameroon 2011*	2394	0.0%	0.0%	5.6%	31.9%	62.4%
Abidjan, Côte d’Ivoire 2011-12*	1137	0.0%	0.2%	9.0%	37.1%	53.7%
Addis Ababa, Ethiopia 2011*	1524	0.3%	0.2%	0.1%	0.4%	98.9%
Kampala, Uganda 2011*	1062	0.0%	0.3%	1.2%	7.7%	90.8%
**P2C wealth quintiles**						
Greater Accra (urban), Ghana 2006	2795	2.2%	7.0%	11.6%	26.8%	52.4%
Ulaanbaatar, Mongolia 2005	9084	6.8%	13.6%	19.7%	25.4%	34.5%
Douala/Yaoundé, Cameroon 2011	2394	1.2%	5.6%	15.9%	27.3%	50.0%
Abidjan, Côte d’Ivoire 2011-12	1137	1.5%	6.3%	19.0%	31.4%	41.7%
Addis Ababa, Ethiopia 2011	1524	0.2%	1.3%	3.2%	11.2%	84.1%
Kampala, Uganda 2011	1062	5.2%	9.0%	13.3%	19.8%	52.6%

DHS – demographic and health surveys, P2C – polychoric dual-component wealth index, n – sample size

*New composite DHS index method (see text for details).

In the case of Kampala, the composite DHS index assigns an unlikely 91% of its population to the highest quintile and less than 2% cumulatively to the three lowest quintiles. The P2C index, on the other hand identifies 5% of Uganda capital’s population as being in the lowest quintile, 9% in the second and 13% in the middle quintiles. Similar comparisons can be made for the other large urban areas shown in **Table 7**. The most extreme case is that of Addis Ababa for which the composite DHS method assigns 99% of the population to the highest quintile, while the approach proposed in this paper assigns 84%; while such a distribution appears unrealistic, the P2C estimates are nevertheless closer to those of Ethiopia’s 2010/11 HCE survey [58], which place 64% of Addis Ababa’s population in the wealthiest national household expenditure quintile.

## DISCUSSION

The detailed Mozambican example helps us appreciate the effects of the various techniques used in the P2C approach, while the additional examples substantiate its relevance to a wider range of data sources.

**Table 3** illustrates how the ambiguously interpretable 2nd second principal component from the PCA of the Mozambican data set gives way to one clearly expressing rural characteristics of wealth once ordered categorical variables are used instead of binary (dummy) variables from a standard DHS approach. This supports the argument of Kolenikov and Angeles [29,30] that the conversion of categorical into indicator variables introduces spurious correlations and, consequently, artificially modifies the orientation of the axes of the principal components. Ordered categorical variables are also best suited to a polychoric PCA.

**Table 4** illustrates how in a polychoric PCA each category of ordinal variables receives a specific coefficient, including the negative answers of yes/no binary variables. As indicated in the Results section, very substantial gains are registered in the proportion of variance explained by the leading principal components when we run a polychoric instead of a standard PCA. Further gains are registered when variables contributing little to the underlying concept of asset-based wealth are dropped by the SMC procedure, in addition to eliminating the noise such inappropriate variables may produce in the results.

**Table 4** also demonstrates how the polychoric results are more coherent than those of the standard PCA. The coefficients of ordered categories consistently progress in the expected direction from the ownership of less valuable to more valuable assets. A key observation is that when taking jointly the coefficients of the first two principal components (adding one to the other), the ownership of each and every binary and continuous asset is associated with a positive overall coefficient, which is not always the case when only the 1st principal component is used. Such a finding lends further support to the theoretical arguments in favour of combining both the 1st and 2nd principal components in the wealth index.

The findings presented in this paper indicate that, at least in the data sets that we analysed that have used the new DHS approach, urban bias continues to be a serious problem. Indeed, some of the DHS surveys that have used this new method openly acknowledge in their report that there is little difference between the wealth indices derived through the traditional DHS method and the new one [57,59,60]. The substantial urban bias persisting in the DHS wealth index is clearly demonstrated in the results of the six large urban centres shown in **Table 7**, particularly the four series related to the surveys using the most recent composite approach for calculating the DHS index: Douala/Yaoundé, Abidjan, Addis Ababa and Kampala. In each of those four large urban populations, the DHS index assigns less than 1% of the population to the lowest two quintiles jointly; even the 3rd quintile is poorly represented. The same table demonstrates how the P2C index assigns more plausible proportions of urban populations to those quintiles. In the case of Maputo, the capital of Mozambique, **Figure 4** demonstrates how, at the population level, the P2C index is in good agreement with expenditure data from the same year, in sharp contrast with the DHS index, suggesting that the proposed approach largely redressed the urban bias. These results argue against the claim that is sometimes made that the reason the DHS index often finds very small proportions of poor in large cities is because in low and middle income countries the poorest urban dwellers generally tend to be better off than most people in rural areas [61]. We rather believe that the substantial underestimation of the proportion of urban poor using the DHS approach essentially reflects a problem with the methodology, not the lack of poor in urban settings.

While this paper does not address policy implications in detail, the results presented in **Table 6** suggest that with respect to at least some indicators the DHS index tends to over-estimate differences between rich and poor, as it confounds urbanicity with wealth. Consequently, using a wealth index that is less biased than the one currently used has potentially important implications for policy makers, particularly given the worldwide attention on reducing inequalities in the pursuit of the SDGs.

This study is subject to a number of limitations. Overall, more extensive analyses, using larger numbers of data sets from different socio-economic contexts, are required to further evaluate the P2C approach and better understand the implications for evidence-based policy that might derive from using an improved, less (urban) biased, measure of socio-economic status as proposed here. Further comparisons of the P2C results with expenditure and/or income measures of socio-economic status are also warranted.

Some details of the proposed approach also deserve additional research. These include experimentation to determine how sensitive PCA results are to different choices of cut-off for the SMC procedure. While a number of papers have already been published on the use of a polychoric PCA for wealth indices, further studies will help improve our understanding of its benefits and potential disadvantages. Last but not least, more research is required into the benefits of using two, possibly more, principal components in the calculation of asset-based wealth indices, as the key technical innovation proposed by the P2C approach.

The presumption, in the case of an asset-based wealth index, is that the variables included in the analysis are directly related to wealth through their investment cost. However, it can be argued that other unmeasured factors influence the acquisition of assets such as availability, accessibility, convenience, and the like. The method proposed in this paper does not directly account for such factors.

## CONCLUSIONS

Equity is receiving increasing attention from public institutions, even more so since the adoption of the sustainable development goals. Producing valid assessments of equity obviously requires valid measures of socio-economic status. We have presented theoretical arguments and practical examples to support our contention that the approach currently widely used to calculate asset-based wealth indices has important limitations, with a strong bias towards urban patterns of wealth. We propose an alternative approach which differs from the standard one in four respects. First, instead of replacing each categorical variable with several binary dummy variables, it requires ordered categorical variables, where applicable, to be used in the analysis. Second, our approach uses a polychoric PCA rather than the standard PCA based on Pearson correlation coefficients. Third, we use an SMC procedure to filter out variables contributing little to the characterization of asset-based wealth. Fourth, our approach combines the first two principal components rather than using only the first principal component.

We have shown that the polychoric dual-component method can be easily applied in mainstream surveys such as the MICS and DHS, as well as in epidemiological studies, and that it yields credible results. Indeed, we believe that the polychoric dual-component approach produces more credible distributions of wealth for the rural and urban subpopulations than either the standard or the composite DHS index methods. We believe the improved performance of the method proposed in this paper results from the elimination of spurious correlations produced by the use dummy variables, the additional information included in the ordinal variables, the better correlation estimates produced by the polychoric approach, the elimination of irrelevant variables through the SMC procedure, and, last but not least, the additional information provided by the 2nd principal component.

## Additional material

Online Supplementary Document

## References

[1] Equity Case Studies | Introduction | UNICEF. 2016. Available: http://www.unicef.org/equity/index_62099.html. Accessed: 6 January 2021.

[2] World Bank Annual Report 2015. Washington: World Bank, 2015.

[3] Countdown to. 2015: A Decade of Tracking Progress for Maternal, Newborn and Child Survival. The 2015 Report. Geneva: UNICEF and WHO, 2015. 10.1016/S0140-6736(15)00519-X PMC7613171 26477328

[4] Barros AJ Ronsmans C Axelson H Loaiza E Bertoldi AD Franca GV Equity in maternal, newborn, and child health interventions in Countdown to 2015: a retrospective review of survey data from 54 countries. Lancet. 2012;379:1225-33. 10.1016/S0140-6736(12)60113-5 22464386

[5] Sustainable Development Goals (SDGs) | UNDP. 2016. Available: http://www.undp.org/content/undp/en/home/sdgoverview/post-2015-development-agenda.html. Accessed: 6 January 2021.

[6] Morris SS Carletto C Hoddinott J Christiaensen LJM Validity of rapid estimates of household wealth and income for health surveys in rural Africa. J Epidemiol Community Health. 2000;54:381-7. . 10.1136/jech.54.5.381 10814660 PMC1731675

[7] Neubourg Cd, Chai J. Milliano Md, Plavgo I, Mei Z. Step-by-step Guidelines to the Multiple Overlapping Deprivation Analysis (MODA). Working Paper. Florence: UNICEF Office of Research, 2012.

[8] Vyas S Kumaranayake L Constructing socio-economic status indices: how to use principal components analysis. Health Policy Plan. 2006;21:459-68. 10.1093/heapol/czl029 17030551

[9] Poirier MJP Grépin KA Grignon M Approaches and Alternatives to the Wealth Index to Measure Socioeconomic Status Using Survey Data: A Critical Interpretive Synthesis. Soc Indic Res. 2020;148:1-46. 10.1007/s11205-019-02187-9

[10] Filmer D Pritchett L Estimating Wealth Effects without Expenditure Data or Tears: With an Application to Educational Enrollments in States of India. SSRN, 1998. 10.1353/dem.2001.0003 11227840

[11] Filmer D Pritchett LH Estimating wealth effects without expenditure data - or tears: an application to educational enrollments in states of India. Demography. 2001;38:115-32. 11227840 10.1353/dem.2001.0003

[12] Rutstein SO, Johnson K. The DHS Wealth Index. DHS Comparative Reports No 6. 2004. 2010-01-20. Available: http://www.measuredhs.com/pubs/pdf/CR6/CR6.pdf. Accessed: 6 January 2021.

[13] UNICEF. MICS3 tools. 2005. Available: http://mics.unicef.org/tools?round=mics3. Accessed: 6 January 2021.

[14] Wagstaff A Watanabe N What difference does the choice of SES make in health inequality measurement? Health Econ. 2003;12:885-90. 10.1002/hec.805 14508873

[15] Hancioglu A. Performance of alternative approaches for identifying the relatively poor and linkages to reproductive health. CICRED Seminar on “Reproductive Health, Unmet Needs, and Poverty: Issues of Access and Quality of Services”; Bangkok, 2002.

[16] DHS and World Bank use wealth index to measure socioeconomic status. DHS+ Dimensions. 2002.

[17] Joseph G Silva I Fink GJD Barros A Victora C Absolute income is a better predictor of coverage by skilled birth attendance than relative wealth quintiles in a multicountry analysis: Comparison of 100 low- and middle-income countries. BMC Pregnancy Childbirth. 2018;18:104. 10.1186/s12884-018-1734-0 29661161 PMC5902965

[18] Howe LD Hargreaves JR Gabrysch S Huttly SR Is the wealth index a proxy for consumption expenditure? A systematic review. J Epidemiol Community Health. 2009;63:871-7. 10.1136/jech.2009.088021 19406742

[19] Howe LD Galobardes B Matijasevich A Gordon D Johnston D Onwujekwe O Measuring socio-economic position for epidemiological studies in low- and middle-income countries: a methods of measurement in epidemiology paper. Int J Epidemiol. 2012;41:871-86. 10.1093/ije/dys037 22438428 PMC3396323

[20] Filmer D Scott K Assessing Asset Indices. Demography. 2012;49:359-92. 10.1007/s13524-011-0077-5 22135117

[21] Sharker MY Nasser M Abedin J Arnold B Luby S The risk of misclassifying subjects within principal component based asset index. Emerg Themes Epidemiol. 2014;11:6. 10.1186/1742-7622-11-6 24987446 PMC4075602

[22] Lattin JM, Carroll JD, Green PE. Analyzing multivariate data. Pacific Grove, CA: Thomson Brooks/Cole; 2003.

[23] StataCorp. Stata: Release 14: Multivariate Statistics [MV]. College Station: Stata Press; 2015.

[24] Van Belle G, Fisher L. Biostatistics: a methodology for the health sciences. 2nd ed. Hoboken, NJ: John Wiley & Sons; 2004.

[25] Hancioglu A. Performance of alternative approaches for identifying the relatively poor and linkages to reproductive health. CICRED Seminar on “Reproductive Health, Unmet Needs, and Poverty: Issues of Access and Quality of Services”; Bangkok2002.

[26] Ward P Measuring the Level and Inequality of Wealth: An Application to China. Rev Income Wealth. 2014;60:613-35. 25641989 10.1111/roiw.12063 PMC4308814

[27] Rutstein SO. Steps to constructing the new DHS Wealth Index. Available: https://dhsprogram.com/programming/wealth%20index/Steps_to_constructing_the_new_DHS_Wealth_Index.pdf. Accessed: 8 February 2020.

[28] DHS. DHS Wealth Index Construction. Available: https://dhsprogram.com/programming/wealth%20index/DHS_Wealth_Index_Files.pdf. Accessed: 8 February 2020.

[29] Kolenikov S, Angeles G. The use of discrete data in PCA: Theory, simulations, and applications to Socioeconomic indices. CPC / MEASURE Working Paper No. WP-04-85. 2004.

[30] Kolenikov S Angeles G Socioeconomic status measurement with discrete proxy variables: Is principal component analysis a reliable answer? Rev Income Wealth. 2009;55:128-65. 10.1111/j.1475-4991.2008.00309.x

[31] Pearson K Mathematical Contributions to the Theory of Evoluation. VII On the Correlation of Characters not Quantitatively Measurable. Philos Trans R Soc Lond. 1900;195:1-47.

[32] Olsson U Maximum likelihood estimation of the polychoric correlation coefficient. Psychometrika. 1979;44:443-60. 10.1007/BF02296207

[33] Pearson K Pearson ES On Polychoric Coefficients of Correlation. Biometrika. 1922;14:127-56. 10.1093/biomet/14.1-2.127

[34] Reidpath DD Ahmadi K A novel nonparametric item response theory approach to measuring socioeconomic position: a comparison using household expenditure data from a Vietnam health survey, 2003. Emerg Themes Epidemiol. 2014;11:9. 10.1186/1742-7622-11-9 25126103 PMC4132525

[35] Howe LD Hargreaves JR Ploubidis GB De Stavola BL Huttly SRA Subjective measures of socio-economic position and the wealth index: a comparative analysis. Health Policy Plan. 2011;26:223-32. 10.1093/heapol/czq043 20817696

[36] Rutstein SO. The DHS wealth index: Approaches for rural and urban areas. DHS Working Papers No 60. 2008.

[37] Montgomery MR Hewett PC Urban poverty and health in developing countries: Household and neighborhood effects. Demography. 2005;42:397-425. 10.1353/dem.2005.0020 16235606

[38] Gwatkin DR, Rutstein S, Johnson K, Suliman E, Wagstaff A, Amouzou A. Socio-economic differences in health, nutrition, and population within developing countries: An overview. Country reports on HNP and poverty. Washington: World Bank, 2007. Report No.: 48361. 18293634

[39] StataCorp. Stata: Release 14. 14.0 ed. College Station, TX 2015.

[40] Greene WH. Econometric Analysis. 7th ed. University NY, editor. New York: Prentice Hall; 2012.

[41] Kolenikov S, Angeles G. The use of discrete data in PCA: Theory, simulations, and applications to Socioeconomic indices. CPC/MEASURE Working Paper No WP-04-85. 2004 17 November 2010.

[42] Jackson JE. A User's Guide to Principal Components. Wiley-interscience, editor. Hoboken (NJ): John Wiley & Sons; 2003.

[43] Moçambique: Inquérito de Indicadores Múltiplos 2008. Maputo: Instituto Nacional de Estatística, 2009.

[44] Moçambique: Inquérito Demográfico e de Saúde 2003. Maputo: Institute Nacional de Estatística, Ministério de Saúde, Measure DHS+/ORC Macro, 2005.

[45] Moçambique: Inquérito Demográfico e de Saúde 1997. Maputo: Instituto Nacional de Estatística, Macro International Inc., 1998.

[46] Moçambique: Inquérito Demográfico e de Saúde 2011. Maputo: Institute Nacional de Estatística, Ministério de Saúde, Measure DHS/ICF International, 2013.

[47] Poverty and wellbeing in Mozambique: Third national poverty assessment. Maputo: Ministry of Planning and Development, DNEAP, 2010.

[48] Manual do Inquiridor IOF. Maputo: Instituto Nacional de Estatísticas; 2008.

[49] Nyandiko WM Mwangi A Ayaya SO Nabakwe EC Tenge CN Gisore PM Characteristics of HIV-infected children seen in Western Kenya. East Afr Med J. 2009;86:364-73. 20575310 10.4314/eamj.v86i8.54156

[50] Ghana: Multiple Indicators Cluster Survey 2006. Accra: Statistical Service Ghana, 2007.

[51] Vietnam: Multiple Indicators Cluster Survey 2006. Hanoi: General Statistics Office of Vietnam, 2007.

[52] Mongolia: Child and development 2005 survey (MICS-3). Ulaanbaatar: National Statistical Office, 2007.

[53] Albania: Multiple Indicator Cluster Survey 2005. Tirana: Albanian National Institute of Statistics, 2008.

[54] Enquête Démographique et de Santé et à Indicateurs Multiples (EDS-MICS) - Cameroun 2011. Yaoundé: Institut National de la Statistique, ICF International, 2012.

[55] Ethiopia Demographic and Health Survey 2011. Addis Ababa: Central Statistical Agency, ICF International, 2012.

[56] Uganda Demographic and Health Survey 2011. Kampala: Uganda Bureau of Statistics, MEASURE DHS, ICF International, 2012.

[57] Enquête Démographique et de Santé et à Indicateurs Multiples (EDS-MICS) - République de Côte d'Ivoire 2011-2012. Abidjan: Institut National de la Statistique, MEASURE DHS, ICF International, 2013.

[58] The 2010/11 Ethiopian Households Consumption - Expenditure (HCE) Survey: Analytical report. Ethiopia Central Statistical Agency, 2012.

[59] Enquête Mortalité, Morbidité et Utilisation des Services (EMMUS-V) - Haiti 2012. Pétion-Ville: Institut Haïtien de l'Enfance, MEASURE DHS, ICF International, 2013.

[60] Howe L. The wealth index as a measure of socio-economic position [PhD]. London: London School of Hygiene and Tropical Medicine; 2009.

[61] Rutstein SO. The DHS wealth index: Approaches for rural and urban areas. DHS Working Papers No. 60. Calverton: Measure DHS, 2008.

