# Supplementary material for: The polychoric dual-component wealth index as an alternative to the DHS index: Addressing the urban bias
Source: J Glob Health. 2021 Jan 30;11:04003. doi: 10.7189/jogh.11.04003 (PMC7897450; doi:10.7189/jogh.11.04003)
Supplement: Online Supplementary Document [file jogh-11-04003-s001.zip › Table S2.docx]

Table S2 Distribution of urban and rural populations into (original) DHS and P2C wealth quintile — 4 countries.

| **Ghana 2006** |  |
| --- | --- |
| \| **DHS index** \| **Distribution of population**  **(1^st^ quintile: poorest)** \| \| \| \| \| \| --- \| --- \| --- \| --- \| --- \| --- \| \| **1^st^** \| **2^nd^** \| **3^rd^** \| **4^th^** \| **5^th^** \| \| **Urban** \| 1% \| 5% \| 19% \| 32% \| 43% \| \| **Rural** \| 33% \| 30% \| 21% \| 12% \| 4% \| \| **Overall** \| 20% \| 20% \| 20% \| 20% \| 20% \| | \| **P2C index** \| **Distribution of population**  **(1^st^ quintile: poorest)** \| \| \| \| \| \| --- \| --- \| --- \| --- \| --- \| --- \| \| **1^st^** \| **2^nd^** \| **3^rd^** \| **4^th^** \| **5^th^** \| \| **Urban** \| 9% \| 13% \| 16% \| 26% \| 37% \| \| **Rural** \| 28% \| 25% \| 23% \| 16% \| 8% \| \| **Overall** \| 20% \| 20% \| 20% \| 20% \| 20% \| |
| **Vietnam 2006** |  |
| \| **DHS index** \| **Distribution of population**  **(1^st^ quintile: poorest)** \| \| \| \| \| \| --- \| --- \| --- \| --- \| --- \| --- \| \| **1st** \| **2nd** \| **3rd** \| **4th** \| **5^th^** \| \| **Urban** \| 3% \| 4% \| 7% \| 19% \| 67% \| \| **Rural** \| 20% \| 23% \| 25% \| 24% \| 9% \| \| **Overall** \| 15% \| 18% \| 21% \| 22% \| 23% \| | \| **P2C index** \| **Distribution of population**  **(1^st^ quintile: poorest)** \| \| \| \| \| \| --- \| --- \| --- \| --- \| --- \| --- \| \| **1st** \| **2nd** \| **3rd** \| **4th** \| **5th** \| \| **Urban** \| 5% \| 9% \| 14% \| 26% \| 46% \| \| **Rural** \| 25% \| 24% \| 22% \| 18% \| 11% \| \| **Overall** \| 20% \| 20% \| 20% \| 20% \| 20% \| |
| **Mongolia 2005** |  |
| \| **DHS index** \| **Distribution of population**  **(1^st^ quintile: poorest)** \| \| \| \| \| \| --- \| --- \| --- \| --- \| --- \| --- \| \| **1st** \| **2nd** \| **3rd** \| **4th** \| **5th** \| \| **Urban** \| 1% \| 12% \| 24% \| 30% \| 34% \| \| **Rural** \| 45% \| 31% \| 15% \| 7% \| 2% \| \| **Overall** \| 20% \| 20% \| 20% \| 20% \| 20% \| | \| **P2C index** \| **Distribution of population**  **(1^st^ quintile: poorest)** \| \| \| \| \| \| --- \| --- \| --- \| --- \| --- \| --- \| \| **1st** \| **2nd** \| **3rd** \| **4th** \| **5th** \| \| **Urban** \| 9% \| 17% \| 19% \| 23% \| 31% \| \| **Rural** \| 34% \| 25% \| 21% \| 16% \| 5% \| \| **Overall** \| 20% \| 20% \| 20% \| 20% \| 20% \| |
| **Albania 2005** |  |
| \| **DHS index** \| **Distribution of population**  **(1^st^ quintile: poorest)** \| \| \| \| \| \| --- \| --- \| --- \| --- \| --- \| --- \| \| **1st** \| **2nd** \| **3rd** \| **4th** \| **5th** \| \| **Urban** \| 2% \| 7% \| 17% \| 30% \| 44% \| \| **Rural** \| 32% \| 28% \| 22% \| 13% \| 4% \| \| **Overall** \| 20% \| 20% \| 20% \| 20% \| 20% \| | \| **P2C index** \| **Distribution of population**  **(1^st^ quintile: poorest)** \| \| \| \| \| \| --- \| --- \| --- \| --- \| --- \| --- \| \| **1st** \| **2nd** \| **3^rd^** \| **4th** \| **5th** \| \| **Urban** \| 14% \| 11% \| 22% \| 28% \| 25% \| \| **Rural** \| 24% \| 27% \| 18% \| 15% \| 17% \| \| **Overall** \| 20% \| 20% \| 20% \| 20% \| 20% \| |

DHS: Demographic and Health Survey; P2C: Polychoric Dual-Component Index
