# Supplementary material for: The polychoric dual-component wealth index as an alternative to the DHS index: Addressing the urban bias
Source: J Glob Health. 2021 Jan 30;11:04003. doi: 10.7189/jogh.11.04003 (PMC7897450; doi:10.7189/jogh.11.04003)
Supplement: Online Supplementary Document [file jogh-11-04003-s001.zip › Table S1.docx]

Table S1 Changes in the distribution of the population by (original) DHS and P2C wealth quintile, and scree plots — 4 countries.

| **Ghana 2006** |  |
| --- | --- |
| \| **DHS idx** \| **P2C index (quintiles)** \| \| \| \| \| \| --- \| --- \| --- \| --- \| --- \| --- \| \| **1^st^**  **^(poorest)^** \| **2^nd^** \| **3^rd^** \| **4^th^** \| **5^th^** \| \| **1^st^** \| 42% \| 27% \| 18% \| 10% \| 4% \| \| **2^nd^** \| 37% \| 30% \| 21% \| 10% \| 2% \| \| **3^rd^** \| 20% \| 35% \| 31% \| 12% \| 2% \| \| **4^th^** \| 2% \| 9% \| 28% \| 46% \| 16% \| \| **5^th^** \| 0% \| 0% \| 2% \| 23% \| 75% \| | 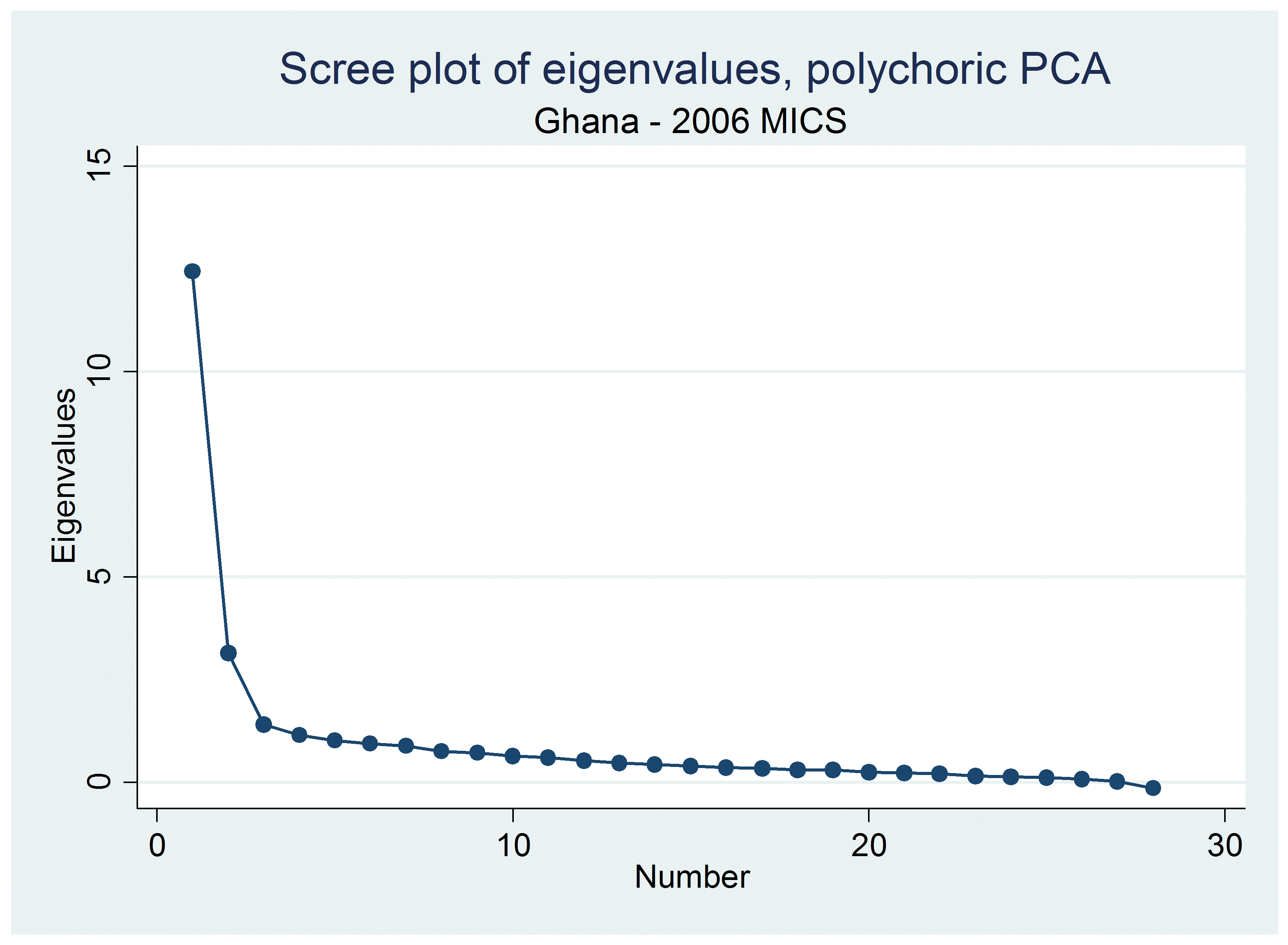 |
| **Vietnam 2006** |  |
| \| **DHS idx** \| **P2C index (quintiles)** \| \| \| \| \| \| --- \| --- \| --- \| --- \| --- \| --- \| \| **1^st^**  **^(poorest)^** \| **2^nd^** \| **3^rd^** \| **4^th^** \| **5^th^** \| \| **1^st^** \| 69% \| 22% \| 6% \| 3% \| 0% \| \| **2^nd^** \| 35% \| 37% \| 21% \| 7% \| 1% \| \| **3^rd^** \| 14% \| 34% \| 33% \| 16% \| 3% \| \| **4^th^** \| 2% \| 13% \| 30% \| 40% \| 15% \| \| **5^th^** \| 0% \| 0% \| 6% \| 26% \| 67% \| | 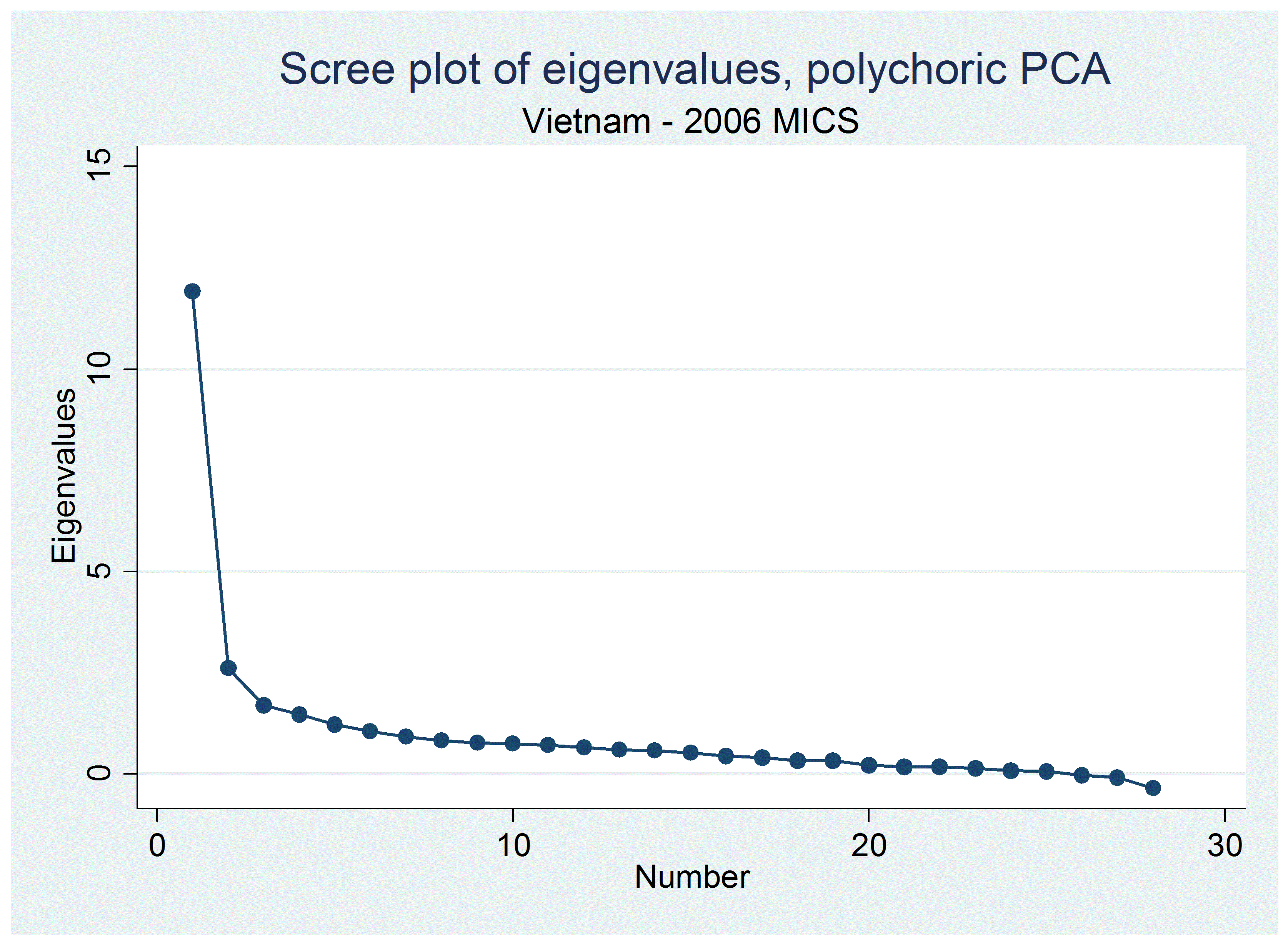 |
| **Mongolia 2005** |  |
| \| **DHS idx** \| **P2C index (quintiles)** \| \| \| \| \| \| --- \| --- \| --- \| --- \| --- \| --- \| \| **1^st^**  **^(poorest)^** \| **2^nd^** \| **3^rd^** \| **4^th^** \| **5^th^** \| \| **1^st^** \| 60% \| 22% \| 9% \| 6% \| 2% \| \| **2^nd^** \| 32% \| 34% \| 21% \| 10% \| 3% \| \| **3^rd^** \| 7% \| 40% \| 38% \| 13% \| 2% \| \| **4^th^** \| 0% \| 5% \| 31% \| 60% \| 4% \| \| **5^th^** \| 0% \| 0% \| 0% \| 11% \| 89% \| | 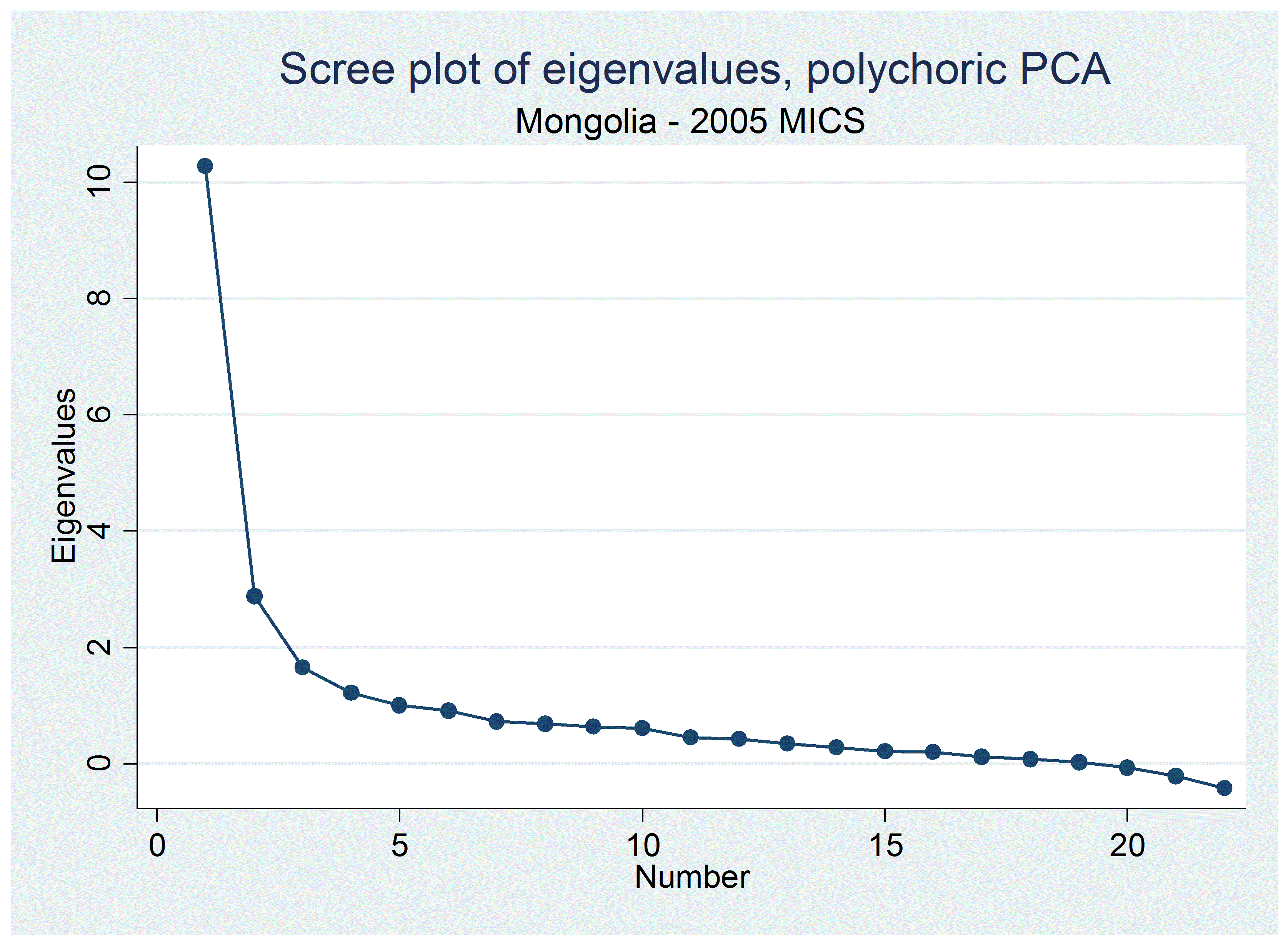 |
| **Albania 2005** |  |
| \| **DHS idx** \| **P2C index (quintiles)** \| \| \| \| \| \| --- \| --- \| --- \| --- \| --- \| --- \| \| **1^st^**  **^(poorest)^** \| **2^nd^** \| **3^rd^** \| **4^th^** \| **5^th^** \| \| **1^st^** \| 45% \| 36% \| 12% \| 5% \| 1% \| \| **2^nd^** \| 26% \| 33% \| 17% \| 15% \| 10% \| \| **3^rd^** \| 17% \| 17% \| 26% \| 23% \| 17% \| \| **4^th^** \| 9% \| 13% \| 20% \| 24% \| 34% \| \| **5^th^** \| 3% \| 4% \| 23% \| 32% \| 38% \| | 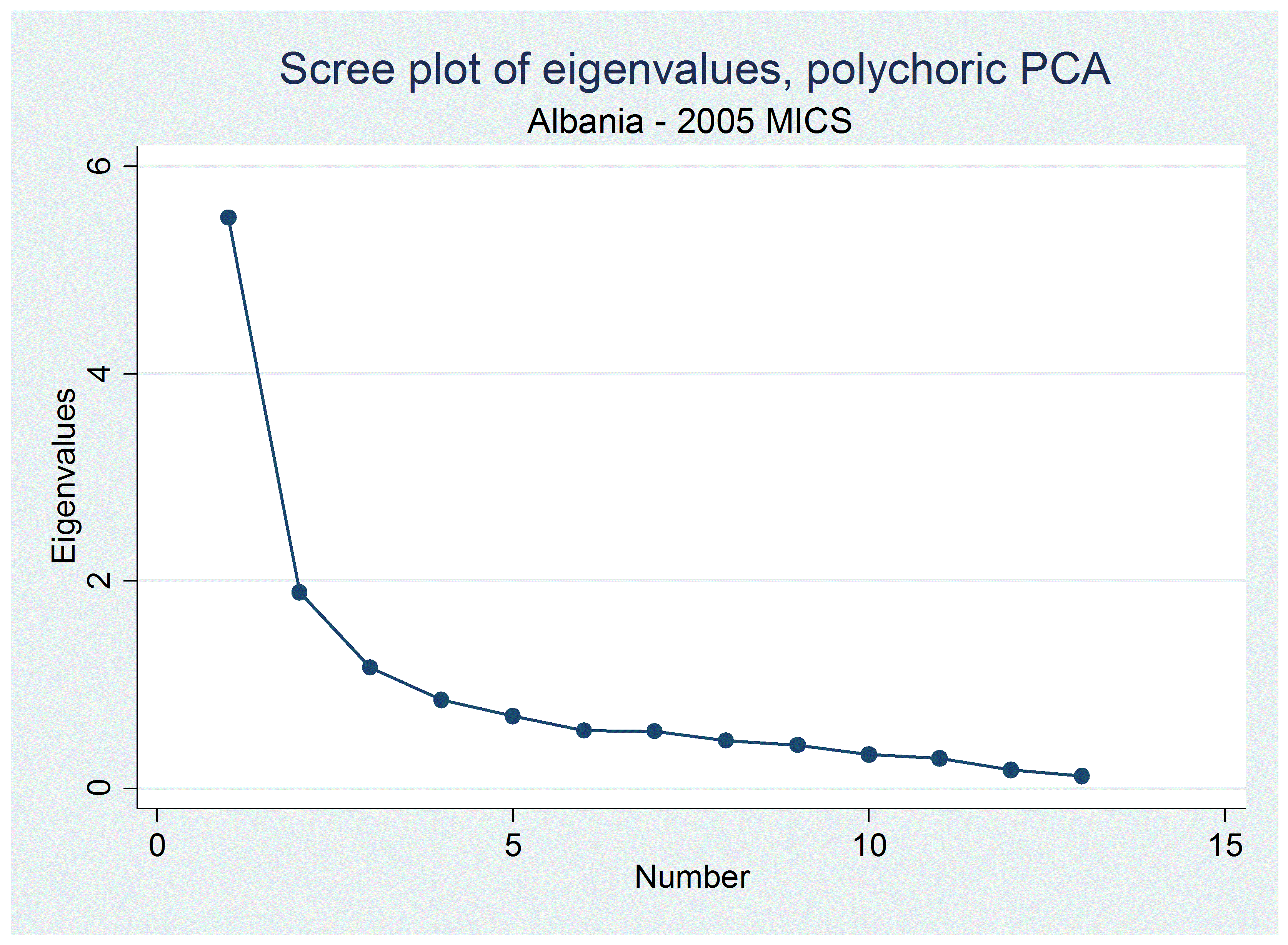 |

DHS: Demographic and Health Survey; P2C: Polychoric Dual-Component Index
